# Supplementary material for: Dietary Factors and Breast Cancer Prognosis among Breast Cancer Survivors: A Systematic Review and Meta-Analysis of Cohort Studies
Source: Cancers (Basel). 2021 Oct 23;13(21):5329. doi: 10.3390/cancers13215329 (PMC8582373; doi:10.3390/cancers13215329)
Supplement: Supplementary file 1 [file cancers-13-05329-s001.zip › cancers-1391014-supplementary.pdf]

cancers

MDPI

Supplementary Materials: Dietary Factors and Breast Cancer Prognosis among Breast Cancer Survivors: A Systematic Review and Meta-Analysis of Cohort Studies

Sin-Hye Park [1,2], Tung Hoang [2] and Jeongseon Kim [2,*]

Sin-Hye Park, Tung Hoang and Jeongseon Kim

Table S1. Baseline characteristic of studies included in the final systematic review and meta-analysis.

Study	Project (follow-up period)	Countries	Diagnostic	Menopausal status	BC stage	Age	Sample size (n)	Exposures	Outcomes	HR/RR (95% CIs)	Covariates
1991 Ewertz M	Danish Breast Cancer Co-operative Group (DBCG;1983-1984 through 1990)	Denmark	Prediagnostic	Premenopausal + Postmenopausal	I-III	< 70 years	1,744 (BC-specific death =805)	Fat consumption (Q4 vs. Q1)	Breast cancer-specific mortality	0.96 (0.75 to 1.22)	Demographic, clinical, reproductive, anthropometric, dietary, age, area of residence
								Alcohol consumption (> 121 g/week vs. 0 g/week)		1.26 (0.90 to 1.74)	
1993 Rohan TE	South Australian Central Cancer Registry (SACCR; median 5.5 years)	Australia	Prediagnostic	Premenopausal + Postmenopausal	-	Mean 55.1 years	412 (BC-specific death =112)	Fat (>108 g/day vs. <56 g/day)	Breast cancer-specific mortality	1.40 (0.66 to 2.96)	Energy, age at menarche, quetelet index
								Protein (>103 g/day vs. <59 g/day)		0.74 (0.34 to 1.66)	
								Carbohydrate (>256 g/day vs. <144 g/day)		0.98 (0.53 to 1.81)	
								Saturated fat (> 45 g/day vs. <20 g/day)		1.65 (0.73 to 3.75)	
								Monounsaturated fat (>37 g/day vs. <17 g/day)		1.33 (0.56 to 3.13)	

1.63 (0.87 to 3.03)

Study	Project (follow-up period)	Countries	Diagnostic	Menopausal status	BC stage	Age	Sample size (n)	Exposures	Outcomes	HR/RR (95% CIs)	Covariates
								Retinol (>1,555 µg/day vs. <250 µg/day)		1.05 (0.56 to 1.95)	
								beta-Carotene (>8,058 µg/day vs. 3,051 µg/day)		0.96 (0.53 to 1.73)	
								Vitamin C (>234 mg/day vs. <71 mg/day)		0.80 (0.47 to 1.38)	
								Alcohol consumption (>10 g/day vs. 0 g/day		1.61 (1.03 to 2.52)	
1994 Jain M	National Breast Screening Study (NBSS; mean 7.7 years)	Canada	Prediagnostic	Premenopausal + Postmenopausal	Invasive BC	Mean 49.8 years	673 (BC-specific death =76)	Saturated fat (>42.94 g/day vs. <26.36 g/day)	Breast cancer-specific mortality	1.91 (0.73 to 5.02)	Total energy, age at diagnosis, smoking, body weight
								Saturated fat/total fat (>41% vs. <35.7%)		1.93 (1.00 to 3.74)	
								Vitamin A supplements (>131,135 IU vs. <6,489 IU)		0.56 (0.28 to 1.09)	
								beta-Carotene (>7,690 IU vs. <3,607 IU)		0.48 (0.23 to 0.99)	
								Vitamin C supplements (>210.3 mg/day vs. <110.7 mg/day)		0.43 (0.21 to 0.86)	

								Vitamin E supplements (>24.33 mg/day vs. <13.73 mg/day)		0.55 (0.26 to 1.17)	
								Thiamin supplements (>1.49 mg/day vs. <0.99 mg/day)		0.57 (0.26 to 1.25)	
								Calcium supplements (>1,005.6 mg/day vs. <541.2 mg/day)		0.66 (0.33 to 1.31)	
				Premenopausal			250 (BC-specific death	Saturated fat (>42.94 g/day vs. <26.36 g/day)		1.14 (0.75 to 1.73)	
Study	Project (follow-up period)	Countries	Diagnostic	Menopausal status	BC stage	Age	Sample size (n)	Exposures	Outcomes	HR/RR (95% CIs)	Covariates
								Saturated fat/total fat (>41% vs. <35.7%)		1.25 (0.47 to 3.31)	
							=26)	Vitamin A supplements (4000 IU/day)		0.78 (0.54 to 1.17)	
								beta-Carotene (2000 IU/day)		0.87 (0.65 to 1.18)	
								Vitamin C supplements (100 mg/day)		0.54 (0.30 to 0.98)	

Study	Project (follow-up period)	Countries	Diagnostic	Menopausal status	BC stage	Age	Sample size (n)	Exposures	Outcomes	HR/RR (95% CIs)	Covariates
								Vitamin E supplements (10 mg/day)		0.64 (0.33 to 1.26)	
								Thiamin supplements (10 mg/day)		1.20 (1.07 to 3.66)	
								Calcium supplements (400 mg/day)		0.93 (0.58 to 1.50)	
				Postmenopausal			423 (BC-specific death =50)	Saturated fat (>42.94 g/day vs. <26.36 g/day)		1.27 (0.94 to 1.73)	
								Saturated fat/total fat (>41% vs. <35.7%)		2.53 (1.20 to 5.33)	
								Vitamin A supplements (4000 IU/day)		0.84 (0.67 to 1.06)	
								beta-Carotene (2000 IU/day)		0.84 (0.68 to 1.03)	
								Vitamin C supplements (100 mg/day)		0.74 (0.50 to 1.11)	
								Vitamin E supplements (10 mg/day)		0.76 (0.51 to 1.13)	
								Thiamin supplements (10 mg/day)		0.62 (0.36 to 1.05)	

								Calcium supplements (400 mg/day)		0.71 (0.49 to 1.05)	
1998 Hebert JR	Memorial Sloan-Kettering Cancer Center (MSK-CC; 1985-1986 through 1991)	United States	Prediagnostic	Premenopausal + Postmenopausal	I-IIIA	Mean 52.2 years	471 (BC-specific death =73; BC recurrence=109)	Red meat	Breast cancer-specific mortality	1.43 (0.74 to 2.79)	Tumor stage, hormone receptor status, age, BMI
								Butter/margarine/lard	Breast cancer-specific mortality	1.16 (0.86 to 1.58)	
								Beer	Breast cancer-specific mortality	1.58 (1.00 to 2.78)	
								Red meat	Breast cancer recurrence	1.12 (0.66 to 1.89)	
								Butter/margarine lard	Breast cancer recurrence	1.30 (1.03 to 1.64)	
								Beer	Breast cancer recurrence	1.41 (1.02 to 1.97)	
				Premenopausal				Meat/liver/bacon	Breast cancer-specific mortality	2.60 (0.96 to 7.03)	
								Butter/margarine/lard	Breast cancer-specific mortality	1.03 (0.61 to 1.76)	
								Beer	Breast cancer-specific mortality	2.33 (1.35 to 4.00)	
								Meat/liver/bacon	Breast cancer recurrence	1.93 (0.89 to 4.15)	
								Butter/margarine/lard	Breast cancer recurrence	1.67 (1.17 to 2.39)	
								Beer	Breast cancer recurrence	1.58 (1.15 to 2.17)	
1999 Holmes MD	Nurses' Health Study (NHS; mean 13.1 years)	United States	Prediagnostic + Postdiagnostic	Premenopausal +Postmenopausal	Invasive BC	Mean 54 years	1,982 (Total death =378; BC-specific death =326)	Poultry (Q4 vs. Q1)	All-cause mortality	0.70 (0.50 to 0.97)	Age, diet interval, calender year of diagnosis, BMI, oral contraceptive use, menopausal status,
								Dairy (Q4 vs. Q1)	All-cause mortality	0.72 (0.52 to 1.00)	
								Fish (Q4 vs. Q1)	All-cause mortality	0.80 (0.60 to 1.07)	
								Vegetables (Q4 vs. Q1)	All-cause mortality	0.81 (0.59 to 1.11)	

Study	Project (follow-up period)	Countries	Diagnostic	Menopausal status	BC stage	Age	Sample size (n)	Exposures	Outcomes	HR/RR (95% CIs)	Covariates
								Fruit (Q4 vs. Q1)		1.07 (0.77 to 1.49)	
								Grain (Q4 vs. Q1)		1.27 (0.90 to 1.79)	
								Red meat (Q4 vs. Q1)		1.06 (0.76 to 1.49)	
								Fiber (Q5 vs. Q1)		0.69 (0.50 to 0.97)	
								Protein (Q5 vs. Q1)		0.65 (0.47 to 0.88)	
								Vitamin A (Q5 vs. Q1)		0.78 (0.58 to 1.06)	
								Lutein/zeaxanthin (Q5 vs. Q1)		0.87 (0.62 to 1.21)	postmenopausal hormone use, smoking, age at first birth and parity, number of metastatic lymph nodes, tumor size, caloric intake
								Calcium (Q5 vs. Q1)		0.66 (0.48 to 0.91)	
								Omega-3 fatty acids (Q5 vs. Q1)		0.77 (0.56 to 1.07)	
								18:2 trans fatty acid (Q5 vs. Q1)		1.45 (1.06 to 1.99)	
								alpha-Carotene (Q5 vs. Q1)		0.93 (0.67 to 1.28)	
								beta-Carotene (Q5 vs. Q1)		0.92 (0.68 to 1.26)	
								beta-Cryptoxanthin (Q5 vs. Q1)		0.86 (0.63 to 1.19)	
								Caffeine (Q5 vs. Q1)		0.77 (0.55 to 1.07)	
								Carotenes (Q5 vs. Q1)		0.96 (0.70 to 1.31)	

Study	Project (follow-up period)	Countries	Diagnostic	Menopausal status	BC stage	Age	Sample size (n)	Exposures	Outcomes	HR/RR (95% CIs)	Covariates
								Folate supplements (Q5 vs. Q1)		0.88 (0.64 to 1.23)	
								Iodine (Q5 vs. Q1)		0.90 (0.67 to 1.21)	
								Iron supplements (Q5 vs. Q1)		0.86 (0.63 to 1.18)	
								Lycopene (Q5 vs. Q1)		0.79 (0.58 to 1.08)	
								Magnesium (Q5 vs. Q1)		0.74 (0.53 to 1.02)	
								Methionine (Q5 vs. Q1)		0.69 (0.50 to 0.94)	
								Niacin (Q5 vs. Q1)		0.80 (0.59 to 1.09)	
								Pantothenic acid (Q5 vs. Q1)		0.92 (0.67 to 1.29)	
								Potassium (Q5 vs. Q1)		0.98 (0.69 to 1.38)	
								Retinol supplements (Q5 vs. Q1)		0.82 (0.59 to 1.13)	
								Selenium (Q5 vs. Q1)		0.86 (0.63 to 1.19)	
								Sodium (Q5 vs. Q1)		0.79 (0.57 to 1.09)	
								Tryptophan (Q5 vs. Q1)		0.63 (0.46 to 0.87)	
								Vitamin B1 supplements (Q5 vs. Q1)		0.82 (0.59 to 1.13)	

Study	Project (follow-up period)	Countries	Diagnostic	Menopausal status	BC stage	Age	Sample size (n)	Exposures	Outcomes	HR/RR (95% CIs)	Covariates
								Vitamin B2 supplements (Q5 vs. Q1)		0.81 (0.58 to 1.13)	
								Vitamin B6 supplements (Q5 vs. Q1)		0.78 (0.56 to 1.09)	
								Vitamin C supplements (Q5 vs. Q1)		1.18 (0.85 to 1.63)	
								Vitamin D supplements (Q5 vs. Q1)		0.86 (0.62 to 1.17)	
								Vitamin E supplements (Q5 vs. Q1)		0.77 (0.56 to 1.05)	
								Zinc supplements (Q5 vs. Q1)		0.83 (0.61 to 1.14)	
			Prediagnostic				1,504 (Total	Fat (Q5 vs. Q1)		1.21 (0.78 to 1.90)	Quantiles of nutrient
							death =238)	Fiber (Q5 vs. Q1)		0.77 (0.45 to 1.25)	or food intake prior to diagnosis, previous diet interval, age, diet interval, calendar year of diagnosis, BMI, oral contraceptive use, menopausal status, postmenopausal hormone use,
								Lutein/zeaxanthin (Q5 vs. Q1)		0.85 (0.53 to 1.38)	
								Calcium (Q5 vs. Q1)		0.64 (0.41 to 0.99)	
								Protein (Q5 vs. Q1)		0.70 (0.46 to 1.08)	
								Omega-3 fatty acids (Q5 vs. Q1)		1.00 (0.62 to 1.60)	
								18:2 trans fatty acid		1.58 (1.03 to 2.43)	

Study	Project (follow-up period)	Countries	Diagnostic	Menopausal status	BC stage	Age	Sample size (n)	Exposures	Outcomes	HR/RR (95% CIs)	Covariates
								(Q5 vs. Q1)			smoking, age at first birth and parity, number of metastatic lymph nodes, tumor size, calories
								Poultry (Q4 vs. Q1)		0.60 (0.39 to 0.92)	
								Dairy (Q4 vs. Q1)		0.71 (0.44 to 1.14)	
								Fish (Q4 vs. Q1)		0.94 (0.62 to 1.43)	
								Vegetables (Q4 vs. Q1)		0.98 (0.62 to 1.53)	
2003 Fleischauer AT	Fatty acid stores tumor characteristics and breast	United States	Prediagnostic	Postmenopausal	I-III	Mean 62.1 years	220 (BC-specific death =23; BC recurrence=41)	Antioxidants supplements (Ever vs. Nonusers)	Breast cancer recurrence or breast cancer-specific mortality	0.54 (0.27 to 1.04)	Age at diagnosis, age at menopause, tumor stage, tamoxifen,
								Vitamin E supplements (Ever vs. Nonusers)		0.55 (0.28 to 1.08)	
	cancer (FASTCAB; 12-14 years)							Vitamin C supplements (Ever vs. Nonusers)		0.64 (0.32 to 1.27)	radiotherapy, HRT, smoking, exercise, dietary intake of vitamin C or E
2004 Borugian MJ	British Columbia Cancer Agency (BCCA; median 8.1 years)	Canada	Prediagnostic	Premenopausal + Postmenopausal	I-III	Mean 54.5 years	603 (BC-specific mortality =91)	Total fat (Q4 vs. Q1)	Breast cancer-specific mortality	1.8 (0.9 to 4.8)	Age, total caloric intake, stage at diagnosis
								Saturated fat (Q4 vs. Q1)		2.5 (1.2 to 5.3)	
								Protein (Q4 vs. Q1)		0.4 (0.2 to 0.8)	
								Total carbohydrates (Q4 vs. Q1)		1.5 (0.7 to 3.4)	
								Fiber (Q4 vs. Q1)		0.7 (0.4 to 1.3)	

								E-Carb (Q4 vs. Q1)		1.7 (0.7 to 3.8)	
				Premenopausal		Mean 43.1 years	235 (BC-specific death =42	Total fat (Q4 vs. Q1)		4.8 (1.3 to 18.1)	
								Saturated fat (Q4 vs. Q1)		4.9 (1.4 to 17.0)	
								Protein (Q4 vs. Q1)		0.2 (0.1 to 0.9)	
								Total carbohydrates (Q4 vs. Q1)		1.3 (0.3 to 5.1)	
								Fiber (Q4 vs. Q1)		0.7 (0.2 to 1.6)	
Study	Project (follow-up period)	Countries	Diagnostic	Menopausal status	BC stage	Age	Sample size (n)	Exposures	Outcomes	HR/RR (95% CIs)	Covariates
								E-Carb (Q4 vs. Q1)		2.1 (0.5 to 8.6)	
				postmenopausal				Total fat (Q4 vs. Q1)		0.7 (0.2 to 2.2)	
								Saturated fat (Q4 vs. Q1)		1.5 (0.5 to 4.0)	
								Protein (Q4 vs. Q1)		0.6 (0.2 to 1.6)	
								Total carbohydrates (Q4 vs. Q1)		2.0 (0.7 to 5.7)	
								Fiber (Q4 vs. Q1)		0.8 (0.3 to 1.8)	
								E-Carb (Q4 vs. Q1)		1.7 (0.6 to 4.9)	
2005 Boyapati SM	Shanghai breast cancer study (SBCS; median 5.2 years)	China	Prediagnostic	Premenopausal +	I-IV	25 to 64 years	1,459 (Total	Total soy protein (T3 vs. T1)	Disease-free survival	0.99 (0.73 to 1.33)	Age at diagnosis, stage of disease, radiotherapy,

				Postmenopausal			death =297)	Total isoflavones (T3 vs. T1)		1.06 (0.79 to 1.42)	ER/PR status, total energy intake
				Premenopausal			339 (Total death =58)	Total soy protein (T3 vs. T1)		1.09 (0.74 to 1.60)	
				Postmenopausal			143 (Total death =39)	Total soy protein (T3 vs. T1)		0.79 (0.49 to 1.28)	
2005 Kroenke CH	Nurses' Health Study (NHS; median 9 years)	United States	Prediagnostic	Premenopausal + Postmenopausal	Invasive BC	Mean 57.8 years	2,619 (Total death =414; BC-specific death =242)	Western diet pattern (Q5 vs. Q1)	All-cause mortality	1.40 (0.93 to 2.09)	Age, BMI, energy intake, smoking, physical activity, diet missing in 1986,

Study	Project (follow-up period)	Countries	Diagnostic	Menopausal status	BC stage	Age	Sample size (n)	Exposures	Outcomes	HR/RR (95% CIs)	Covariates
								Western diet pattern (Q5 vs. Q1)	Breast cancer-specific mortality	1.01 (0.59 to 1.72)	1990, 1994, age at menarche, oral contraceptive use, birth index, menopausal status, use of postmenopausal hormone therapy, age at menopause, tamoxifen use, chemotherapy, stage at diagnosis
			Postdiagnostic					Western diet pattern	All-cause mortality	1.53 (1.03 to 2.29)	Age, time since diagnosis, BMI,

								(Q5 vs. Q1)			energy intake, smoking, physical activity, diet missing in 1986, 1990, 1994, 1998, age a,
								Prudent diet pattern (Q5 vs. Q1)		0.78 (0.54 to 1.12)	
								Western diet pattern (Q5 vs. Q1)	Breast cancer-specific mortality	1.01 (0.60 to 1.70)	
								Prudent diet pattern (Q5 vs. Q1)		1.07 (0.66 to 1.73)	

Study	Project (follow-up period)	Countries	Diagnostic	Menopausal status	BC stage	Age	Sample size (n)	Exposures	Outcomes	HR/RR (95% CIs)	Covariates
											menarche, oral contraceptive use, birth index, menopausal status, use of postmenopausal hormone therapy, age at menopause, tamoxifen, chemotherapy, stage at diagnosis
2006 Cui Y	Shaghai breast cancer study (SBCS; mean 4.8 years)	China	Prediagnostic	Premenopausal + Postmenopausal	I-IV	Mean 48.2 years	1,455 (Total death =191; BC recurrence=235)	Ginseng (Regular use vs. Never use)	All-cause mortality	0.71 (0.52 to 0.98)	Age at diagnosis, marital status, education, income, tumor-node metastasis, ER/PR status, surgery, chemotherapy, radiotherapy, tamoxifen use
								Ginseng (Regular use vs. Never use)	Disease-free survival, recurrence	0.70 (0.53 to 0.93)	

Study	Project (follow-up period)	Countries	Diagnostic	Menopausal status	BC stage	Age	Sample size (n)	Exposures	Outcomes	HR/RR (95% CIs)	Covariates
2006 McEligot AJ	Cancer surveillance program of Orange County (CSPOC; mean 6.7 years)	United States	Prediagnostic	Postmenopausal	*In situ* and metastatic BC	Mean 64.78 years	516 (Total death =96; BC-specific death =41)	Vegetables (T3 vs. T1)	All-cause mortality	0.57 (0.35 to 0.94)	Stage of disease, age at diagnosis, BMI, parity, HRT use, alcohol use, multivitamin use, energy intake
								Fruit (T3 vs. T1)		0.63 (0.38 to 1.05)	
								Total fat (T3 vs. T1)		3.12 (1.79 to 5.44)	
								Saturated fat (T3 vs. T1)		4.45 (2.26 to 8.78)	
								Oleic fatty acid (T3 vs. T1)		3.56 (1.67 to 7.59)	
								Linoleic fatty acid (T3 vs. T1)		2.39 (1.21 to 4.69)	
								Fiber (T3 vs. T1)		0.48 (0.27 to 0.86)	
								Folate (T3 vs. T1)		0.34 (0.18 to 0.67)	
								Vitamin C (T3 vs. T1)		0.45 (0.25 to 0.78)	
								alpha-Carotene (T3 vs. T1)		0.77 (0.45 to 1.30)	
								beta-Carotene (T3 vs. T1)		0.50 (0.29 to 0.85)	
								beta-Cryptoxanthin (T3 vs. T1)		0.54 (0.30 to 0.96)	
								Lutein (T3 vs. T1)		0.50 (0.30 to 0.84)	
								Lycopene (T3 vs. T1)		0.76 (0.45 to 1.30)	
								Provitamin A supplements (T3 vs. T1)		0.58 (0.34 to 0.99)	

								Folate supplements (T3 vs. T1)		1.05 (0.54 to 2.03)	
								Vitamin C supplements (T3 vs. T1)		0.58 (0.31 to 1.09)	

Study	Project (follow-up period)	Countries	Diagnostic	Menopausal status	BC stage	Age	Sample size (n)	Exposures	Outcomes	HR/RR (95% CIs)	Covariates
								beta-Carotene supplements (T3 vs. T1)		0.69 (0.40 to 1.20)	
2006 Fink BN	Long Island breast cancer study project (LIBCSP; 1996-1997 through 2002 to 2004)	United States	Postdiagnostic	Premenopausal	*in situ* or Invasive BC	-	376 (Total death =43)	Any fruits, fruit juices, and vegetables (>46 variables vs. 0-18 variables)	All-cause mortality	1.38 (0.65 to 2.91)	Reproductive and menstrual histories, active and passive smoking, alcohol intake, body size, physical activity, medical history
								Any fruits and fruit juices (>24 variables vs. 0-6 variables)		1.10 (0.48 to 2.52)	
								Citrus fruits (>12 variables vs. 0-1 variables)		1.70 (0.75 to 3.89)	
								Any vegetables (>24 variables vs. 0-8 variables)		1.40 (0.71 to 2.76)	
								Leafy vegetables (>9 variables vs. 0-2 variables)		0.85 (0.39 to 1.85)	
								Yellow vegetables (>16 variables vs. 0-4 variables)		1.09 (0.52 to 2.28)	

Study	Project (follow-up period)	Countries	Diagnostic	Menopausal status	BC stage	Age	Sample size (n)	Exposures	Outcomes	HR/RR (95% CIs)	Covariates
								Cruciferous vegetables (>6 variables vs. 0-1 variables)		0.72 (0.34 to 1.54)	
								Dietary alpha-Carotene (>401.9 μg/day vs. 0-74.5 μg/day)		0.76 (0.35 to 1.67)	
								Dietary beta-Carotene (>3,753.8 μg/day vs. 0-1,067.9 μg/day)		0.82 (0.37 to 1.82)	
								Dietary cryptoxanthin (>133.6 μg/day vs. 0-31.2 μg/day)		1.13 (0.53 to 2.41)	
								Dietary lutein (>3,073.5 μg/day vs. 0-675.6 μg/day)		1.71 (0.89 to 3.29)	
								Dietary lycopene (>2,263.9 μg/day vs. 0-548.6 μg/day)		0.61 (0.29 to 1.29)	
								Dietary vitamin C (>173 mg/day vs. 0-60.7 mg/day)		0.90 (0.42 to 1.94)	
								Dietary vitamin E (>10.4 a-te/day		0.96 (0.44 to 2.09)	

								vs. 0-4.4 a-te/day)			
								Any fruits, fruit juices, and vegetables (>46 variables vs. 0-18 variables)		0.68 (0.42 to 1.09)	
				Postmenopausal			834 (Total death =132)	Any fruits and fruit juices (>24 variables vs. 0-6 variables)		0.87 (0.57 to 1.35)	
								Citrus fruits (>12 variables vs. 0-1 variables)		0.90 (0.56 to 1.44)	
								Any vegetables (>24 variables vs. 0-8 variables)		0.92 (0.57 to 1.48)	
								Leafy vegetables (>9 variables vs. 0-2 variables)		0.72 (0.41 to 1.24)	

Study	Project (follow-up period)	Countries	Diagnostic	Menopausal status	BC stage	Age	Sample size (n)	Exposures	Outcomes	HR/RR (95% CIs)	Covariates
								Yellow vegetables (>16 variables vs. 0-4 variables)		0.90 (0.58 to 1.40)	
								Cruciferous vegetables (>6 variables vs. 0-1 variables)		1.07 (0.67 to 1.72)	
								Dietary alpha-Carotene (>401.9		1.20 (0.79 to 1.81)	

Study	Project (follow-up period)	Countries	Diagnostic	Menopausal status	BC stage	Age	Sample size (n)	Exposures	Outcomes	HR/RR (95% CIs)	Covariates
								µg/day vs. 0-74.5 µg/day)			
								Dietary beta-Carotene (>3,753.8 µg/day vs. 0-1,067.9 µg/day)		1.07 (0.70 to 1.64)	
								Dietary cryptoxanthin (>133.6 µg/day vs. 0-31.2 µg/day)		0.82 (0.53 to 1.28)	
								Dietary lutein (>3,073.5 µg/day vs. 0-675.6 µg/day)		0.68 (0.42 to 1.12)	
								Dietary lycopene (>2,263.9 µg/day vs. 0-548.6 µg/day)		0.79 (0.48 to 1.30)	
								Dietary vitamin C (>173 mg/day vs. 0-60.7 mg/day)		1.08 (0.70 to 1.66)	
								Dietary vitamin E (>10.4 a-te/day vs. 0-4.4 a-te/day)		0.77 (0.47 to 1.27)	

Study	Project (follow-up period)	Countries	Diagnostic	Menopausal status	BC stage	Age	Sample size (n)	Exposures	Outcomes	HR/RR (95% CIs)	Covariates
2007 Fink BN	Long Island breast cancer study project (LIBCSP;	United States	Prediagnostic		Invasive BC	-	1,210 (Total	Total flavonoids (>340.5 mg/day	All-cause mortality	0.96 (0.66 to 1.40)	Age and energy

1996-1997 through 2002 to 2004)		death =173)	vs. 0-42.4 mg/day)	
			Total flavonols (>14.5 mg/day vs. 0-3.4 mg/day)	1.12 (0.78 to 1.62)
			Total flavones (>0.20 mg/day vs. 0-0.03 mg/day)	0.63 (0.41 to 0.96)
			Total flavanones (>48.6 mg/day vs. 0-4.0 md/day)	1.03 (0.72 to 1.48)
	Premenopausal+Postmenopausal		Total flavan-3-ols (>263.8 mg/day vs. 0-5.0 mg/day)	1.01 (0.70 to 1.46)
			Total anthocyanidins (>4.24 mg/day vs. 0-0.03 mg/day)	0.64 (0.42 to 0.98)
			Total isoflavones (>7.48 mg/day vs. 0-0.29 mg/day)	0.52 (0.33 to 0.82)
			Total lignans (>9.0 mg/day vs. 0-2.2 mg/day)	1.03 (0.71 to 1.49)
	Premenopausal	376 (Total death =43)	Total flavonoids (>340.5 mg/day vs. 0-42.4 mg/day)	1.77 (0.91 to 3.46)

Study	Project (follow-up period)	Countries	Diagnostic	Menopausal status	BC stage	Age	Sample size (n)	Exposures	Outcomes	HR/RR (95% CIs)	Covariates
								Total flavonols (>14.5 mg/day vs. 0-3.4 mg/day)		1.64 (0.84 to 3.17)	
								Total flavones (>0.20 mg/day vs. 0-0.03 mg/day)		0.69 (0.32 to 1.47)	
								Total flavanones (>48.6 mg/day vs. 0-4.0 mg/day)		1.08 (0.48 to 2.43)	
								Total flavan-3-ols (>263.8 mg/day vs. 0-5.0 mg/day)		1.76 (0.91 to 3.42)	
								Total anthocyanidins (>4.24 mg/day vs. 0-0.03 mg/day)		0.62 (0.27 to 1.40)	
								Total isoflavones (>7.48 mg/day vs. 0-0.29 mg/day)		0.71 (0.34 to 1.48)	
								Total lignans (>9.0 mg/day vs. 0-2.2 mg/day)		1.27 (0.63 to 2.54)	
				Postmenopausal			834 (Total death =130)	Total flavonoids (>340.5 mg/day vs. 0-42.4 mg/day)		0.78 (0.49 to 1.25)	

Study	Project (follow-up period)	Countries	Diagnostic	Menopausal status	BC stage	Age	Sample size (n)	Exposures	Outcomes	HR/RR (95% CIs)	Covariates
								Total flavonols (>14.5 mg/day vs. 0-3.4 mg/day)		0.98 (0.62 to 1.53)	
								Total flavones (>0.20 mg/day vs. 0-0.03 mg/day)		0.59 (0.35 to 0.99)	
								Total flavanones (>48.6 mg/day vs. 0-4.0 mg/day)		0.99 (0.66 to 1.49)	
								Total flavan-3-ols (>263.8 mg/day vs. 0-5.0 mg/day)		0.84 (0.53 to 1.32)	
								Total anthocyanidins (>4.24 mg/day vs. 0-0.03 mg/day)		0.66 (0.40 to 1.08)	
								Total isoflavones (>7.48 mg/day vs. 0-0.29 mg/day)		0.44 (0.24 to 0.81)	
								Total lignans (>9.0 mg/day vs. 0-2.2 mg/day)		0.98 (0.63 to 1.54)	
				Premenopausal + Postmenopausal			1,148 (BC-specific	Total flavonoids (>340.5 mg/day vs. 0-42.4 mg/day)	Breast cancer-specific mortality	0.88 (0.55 to 1.42)	

Study	Project (follow-up period)	Countries	Diagnostic	Menopausal status	BC stage	Age	Sample size (n)	Exposures	Outcomes	HR/RR (95% CIs)	Covariates
							death =113)	Total flavonols (>14.5 mg/day vs. 0-3.4 mg/day)		1.20 (0.77 to 1.87)	
								Total flavones (>0.20 mg/day vs. 0-0.03 mg/day)		0.48 (0.27 to 0.84)	
								Total flavanones (>48.6 mg/day vs. 0-4.0 mg/day)		0.98 (0.62 to 1.56)	
								Total flavan-3-ols (>263.8 mg/day vs. 0-5.0 mg/day)		0.89 (0.55 to 1.43)	
								Total anthocyanidins (>4.24 mg/day vs. 0-0.03 mg/day)		0.68 (0.41 to 1.13)	
								Total isoflavones (>7.48 mg/day vs. 0-0.29 mg/day)		0.87 (0.54 to 1.41)	
								Total lignans (>9.0 mg/day vs. 0-2.2 mg/day)		0.95 (0.60 to 1.51)	
Study	Project (follow-up period)	Countries	Diagnostic	Menopausal status	BC stage	Age	Sample size (n)	Exposures	Outcomes	HR/RR (95% CIs)	Covariates

			Total flavonoids (>340.5 mg/day vs. 0-42.4 mg/day)	1.75 (0.82 to 3.72)
			Total flavonols (>14.5 mg/day vs. 0-3.4 mg/day)	1.64 (0.78 to 3.46)
			Total flavones (>0.20 mg/day vs. 0-0.03 mg/day)	0.45 (0.17 to 1.19)
	Premenopausal	367 (BC-specific death =34)	Total flavanones (>48.6 mg/day vs. 0-4.0 md/day)	0.61 (0.21 to 1.81)
			Total flavan-3-ols (>263.8 mg/day vs. 0-5.0 mg/day)	1.75 (0.83 to 3.69)
			Total anthocyanidins (>4.24 mg/day vs. 0-0.03 mg/day)	0.81 (0.35 to 1.89)
			Total isoflavones (>7.48 mg/day vs. 0-0.29 mg/day)	1.03 (0.46 to 2.28)

Study	Project (follow-up period)	Countries	Diagnostic	Menopausal status	BC stage	Age	Sample size (n)	Exposures	Outcomes	HR/RR (95% CIs)	Covariates
								Total lignans (>9.0 mg/day vs. 0-2.2 mg/day)		1.16 (0.52 to 2.58)	
				Postmenopausal			781 (BC-specific death =79)	Total flavonoids (>340.5 mg/day vs. 0-42.4 mg/day)		0.62 (0.33 to 1.16)	
								Total flavonols (>14.5 mg/day vs. 0-3.4 mg/day)		1.02 (0.59 to 1.79)	
								Total flavones (>0.20 mg/day vs. 0-0.03 mg/day)		0.49 (0.24 to 0.99)	
								Total flavanones (>48.6 mg/day vs. 0-4.0 md/day)		1.09 (0.65 to 1.82)	
								Total flavan-3-ols (>263.8 mg/day vs. 0-5.0 mg/day)		0.63 (0.34 to 1.18)	
								Total anthocyanidins (>4.24 mg/day vs. 0-0.03 mg/day)		0.62 (0.33 to 1.18)	

								Total isoflavones (>7.48 mg/day vs. 0-0.29 mg/day)		0.79 (0.43 to 1.44)	
								Total lignans (>9.0 mg/day vs. 0-2.2 mg/day)		0.87 (0.49 to 1.55)	
2008 Xu X	Long Island breast cancer study project (LIBCSP; mean 5.6 years)	United States	Prediagnostic	Premenopausal + Postmenopausal	In situ or invasive BC	Mean 58.8 years	1,508 (Total death =198; BC-specific death =124)	Dietary folate (High vs. Low)	All-cause mortality	0.79 (0.52 to 1.12)	Age and energy
Study	Project (follow-up period)	Countries	Diagnostic	Menopausal status	BC stage	Age	Sample size (n)	Exposures	Outcomes	HR/RR (95% CIs)	Covariates
								Total folate (diet + supplements; High vs. Low)		0.97 (0.69 to 1.36)	
								Vitamin B1 (thiamin; High vs. Low)		0.54 (0.38 to 0.88)	
								Vitamin B2 (riboflavin; High vs. Low)		0.92 (0.58 to 1.44)	
								Vitamin B3 (niacin; High vs. Low)		0.61 (0.38 to 0.98)	
								Vitamin B6 (pyridoxine; High vs. Low)		0.95 (0.61 to 1.48)	

Study	Project (follow-up period)	Countries	Diagnostic	Menopausal status	BC stage	Age	Sample size (n)	Exposures	Outcomes	HR/RR (95% CIs)	Covariates
								Vitamin B12 (cobalamin; High vs. Low)		1.20 (0.80 to 1.81)	
								Methionine (High vs. Low)		0.70 (0.44 to 1.13)	
								Betaine (High vs. Low)		0.81 (0.54 to 1.20)	
								Dietary folate (High vs. Low)	Breast cancer-specific mortality	0.81 (0.47 to 1.39)	
								Total folate (diet + supplements; High vs. Low)		1.24 (0.81 to 1.90)	
								Vitamin B1 (thiamin; High vs. Low)		0.44 (0.24 to 0.81)	
								Vitamin B2 (riboflavin; High vs. Low)		0.72 (0.41 to 1.29)	
								Vitamin B3 (niacin; High vs. Low)		0.61 (0.34 to 1.09)	
								Vitamin B6 (pyridoxine; High vs. Low)		0.77 (0.44 to 1.36)	
								Vitamin B12 (cobalamin; High vs. Low)		1.10 (0.65 to 1.85)	
								Methionine (High vs. Low)		0.70 (0.39 to 1.28)	
								Betaine (High vs. Low)		0.72 (0.44 to 1.17)	

Study	Project (follow-up period)	Countries	Diagnostic	Menopausal status	BC stage	Age	Sample size (n)	Exposures	Outcomes	HR/RR (95% CIs)	Covariates
2009 Guha N	Life after cancer epidemiology study (LACE; mean 6.31 years)	United States	Prediagnostic	Premenopausal + Postmenopausal	I-IIIA		1,954 (BC recurrence=282)	Daidzein intake (>9,596.55 μg/day vs. 0 μg/day)	Breast cancer recurrence	0.96 (0.52 to 1.76)	Soy supplement use, BMI 1 year before diagnosis, menopausal status, tobacco pack-years, tumor stage, ER status, age, race, kilocalories
								Genistein intake (>13,025.88 μg/day vs. 0 μg/day)		0.95 (0.52 to 1.75)	
								Glycetin intake (>795.40 μg/day vs. 0-3.61 μg/day)		0.80 (0.42 to 1.50)	
				Premenopausal				Daidzein intake (>9,596.55 μg/day vs. 0 μg/day)		1.74 (0.63 to 4.76)	Soy supplement use, BMI 1 year before diagnosis, tobacco pack-years, tumor stage, ER status, age, race, kilocalories
								Genistein intake (>13,025.88 μg/day vs. 0 μg/day)		1.75 (0.65 to 4.76)	
								Glycetin intake (>795.40 μg/day vs. 0-3.61 μg/day)		1.60 (0.54 to 4.72)	
Study	Project (follow-up period)	Countries	Diagnostic	Menopausal status	BC stage	Age	Sample size (n)	Exposures	Outcomes	HR/RR (95% CIs)	Covariates
				Postmenopausal				Daidzein intake (>9,596.55 μg/day vs. 0 μg/day)		0.70 (0.27 to 1.77)	
								Genistein intake (>13,025.88 μg/day vs. 0 μg/day)		0.69 (0.27 to 1.75)	

Study	Project (follow-up period)	Countries	Diagnostic	Menopausal status	BC stage	Age	Sample size (n)	Exposures	Outcomes	HR/RR (95% CIs)	Covariates
								Glycetin intake (>795.40 μg/day vs. 0-3.61 μg/day)		0.51 (0.18 to 1.38)	
2009 Kwan ML	Life after cancer epidemiology study (LACE; mean 5.93 years)	United States	Prediagnostic	Premenopausal + Postmenopausal	I-IIIA	Mean 58.6 years	1,901 (Total death =213; BC-specific death =121; BC recurrence=256)	Prudent diet pattern (Q4 vs. Q1)	All-cause mortality	0.57 (0.36 to 0.90)	Age at diagnosis, total energy intake (kcal), race, BMI at enrollment, total physical activity, smoking, menopausal status at diagnosis, weight change from before diagnosis to baseline, stage of cancer, hormone receptor status,
								Western diet pattern (Q4 vs. Q1)		1.53 (0.93 to 2.54)	treatment
								Prudent diet pattern (Q4 vs. Q1)	Breast cancer-specific mortality	0.79 (0.43 to 1.43)	
								Western diet pattern (Q4 vs. Q1)		1.20 (0.62 to 2.32)	
								Prudent diet pattern (Q4 vs. Q1)	Breast cancer recurrence	0.95 (0.63 to 1.43)	

								Western diet pattern (Q4 vs. Q1)		0.98 (0.62 to 1.54)	
2009 Shu XO	Shanhai breast cancer survival study (SBCSS; median 3.9 years)	China	Postdiagnostic	Premenopausal + Postmenopausal	I-IV	20 to 75 years	5,042 (Total death =444; BC-specific death or recurrence=534)	Soy protein (>15.31 g/day vs. <5.31 g/day)	All-cause mortality	0.71 (0.54 to 0.92)	Age at diagnosis, TNM stage, chemotherapy, radiotherapy, type of surgery received, BMI, menopausal status, ER/PR status, tamoxifen use, education level, income, cruciferous vegetable intake, total meat intake,
								Isoflavones (>62.68 mg/day vs. <20.00 mg/day)		0.79 (0.61 to 1.03)	
								Soy protein (>15.31 g/day vs. <5.31 g/day)	Breast cancer-specific mortality or recurrence	0.68 (0.54 to 0.87)	
								Isoflavones (>62.68 mg/day vs. <20.00 mg/day)		0.65 (0.51 to 0.84)	
Study	Project (follow-up period)	Countries	Diagnostic	Menopausal status	BC stage	Age	Sample size (n)	Exposures	Outcomes	HR/RR (95% CIs)	Covariates
											vitamin supplement use, tea consumption, physical activity
2010 Hellmann SS	Copenhagen city heart study (CCHS; median 7.8 years)	Denmark	Prediagnostic	Premenopausal + postmenopausal	-	Median 66.9 years	528 (Total death =323)	Alcohol consumption (>14 units/week vs. 1 unit/week)	All-cause mortality	1.06 (0.68 to 1.66)	Alcohol, smoking, physical activity, BMI, HRT, age, disease stage, menopausal status, parity, education, adjuvant treatment
								Alcohol consumption	Breast cancer-specific mortality	1.39 (0.77 to 2.52)	Alcohol, smoking,

								(>14 units/week vs. 1 unit/week)			physical activity, BMI, HRT, age, disease stage, parity, education, adjuvant treatment
				Premenopausal			85 (Total death =31)	Alcohol consumption (>14 units/week vs. 1 unit/week)	All-cause mortality	0.23 (0.03 to 1.54)	
				Postmenopausal			443 (Total death =292)	Alcohol consumption (>14 units/week vs. 1 unit/week)		0.93 (0.57 to 1.50)	
2010 Kang X	Harbin medical university (HMU; median 5.1 years)	China	Prediagnostic	Premenopausal	I-III	29 to 72 years	248 (Total death =76; BC recurrence=94)	Soy isoflavones (>42.3 mg/day vs. <15.2 mg/day)	Breast cancer-specific mortality	1.05 (0.78 to 1.71)	Age at diagnosis, TNM stage, ER/PR status,
Study	Project (follow-up period)	Countries	Diagnostic	Menopausal status	BC stage	Age	Sample size (n)	Exposures	Outcomes	HR/RR (95% CIs)	Covariates
								Soy isoflavones (>42.3 mg/day vs. <15.2 mg/day)	Breast cancer recurrence	0.88 (0.61 to 1.23)	chemotherapy, radiotherapy
				Postmenopausal			276 (Total death =78; BC recurrence=91)	Soy isoflavones (>42.3 mg/day vs. <15.2 mg/day)	Breast cancer-specific mortality	0.88 (0.56 to 1.24)	
								Soy isoflavones (>42.3 mg/day vs. <15.2 mg/day)	Breast cancer recurrence	0.67 (0.54 to 0.85)	
2010 Kwan ML	Life after cancer epidemiology study (LACE; mean 7.42 years)	United States	Postdiagnostic	Premenopausal + Postmenopausal	Invasive BC	18 to 70 years	1,897 (Total death =273; BC-specific	Total alcohol (>6.0 g/day vs. None)	All-cause mortality	1.19 (0.87 to 1.62)	Age at diagnosis, prediagnosis BMI, total folate intake, stage of disease, hormone
								Wine		1.08 (0.77 to 1.52)	

Study	Project (follow-up period)	Countries	Diagnostic	Menopausal status	BC stage	Age	Sample size (n)	Exposures	Outcomes	HR/RR (95% CIs)	Covariates
							death =154; BC recurrence =185)	(>2 servings/week vs. None)			receptor status, tamoxifen use, treatment, positive lymph nodes
								Total alcohol (>6.0 g/day vs. None)	Breast cancer-specific mortality	1.51 (1.00 to 2.29)	
								Wine (>2 servings/week vs. None)		1.37 (0.88 to 2.14)	
								Total alcohol (>6.0 g/day vs. None)	Breast cancer recurrence	1.35 (1.00 to 1.83)	
								Wine (>2 servings/week vs. None)		1.33 (0.97 to 1.81)	
2011 Beasley JM	Collaborative women's longevity study (CWLS;	United States	Postdiagnostic	Premenopausal + Postmenopausal	Invasive BC	20 to 79 years	4,441 (Total death =525; BC-specific death	Total fat (Q5 vs. Q1)	All-cause mortality	1.05 (0.79 to 1.39)	Age, state of residence, menopausal status, smoking,
Study	Project (follow-up period)	Countries	Diagnostic	Menopausal status	BC stage	Age	Sample size (n)	Exposures	Outcomes	HR/RR (95% CIs)	Covariates
	mean 5.5 years)						=137)	Saturated fat (Q5 vs. Q1)		1.41 (1.06 to 1.87)	Breast cancer stage, alcohol, history of HRT, interval between diagnosis and diet assessment, energy intake, breast cancer treatment, BMI, physical activity
								Trans fat (Q5 vs. Q1)		1.78 (1.35 to 2.32)	
								Monounsaturated fat (Q5 vs. Q1)		1.14 (0.86 to 1.52)	
								Polyunsaturated fat (Q5 vs. Q1)		0.91 (0.70 to 1.19)	
								Carbohydrates (Q5 vs. Q1)		0.97 (0.72 to 1.30)	

								Protein (Q5 vs. Q1)		0.98 (0.73 to 1.31)	
								Alcohol (Q5 vs. Q1)		0.78 (0.60 to 1.01)	
								Vitamin A (Q5 vs. Q1)		1.12 (0.84 to 1.50)	
								alpha-Carotene (Q5 vs. Q1)		1.08 (0.81 to 1.43)	
								beta-Carotene (Q5 vs. Q1)		1.17 (0.88 to 1.57)	
								beta-Cryptoxanthin (Q5 vs. Q1)		1.25 (0.93 to 1.68)	
								Lutein/zeaxanthin (Q5 vs. Q1)		1.05 (0.77 to 1.43)	
								Lycopene (Q5 vs. Q1)		1.11 (0.83 to 1.47)	
								Fiber (Q5 vs. Q1)		0.75 (0.52 to 1.09)	
								Whole grain (Q5 vs. Q1)		0.79 (0.59 to 1.08)	
								Calcium (Q5 vs. Q1)		0.74 (0.53 to 1.02)	
								Vitamin D (Q5 vs. Q1)		0.86 (0.64 to 1.16)	
								Dairy (Q4 vs. Q1)		1.18 (0.90 to 1.54)	
								Meat (Q4 vs. Q1)		1.12 (0.83 to 1.51)	
Study	Project (follow-up period)	Countries	Diagnostic	Menopausal status	BC stage	Age	Sample size (n)	Exposures	Outcomes	HR/RR (95% CIs)	Covariates
								Vegetables (Q4 vs. Q1)		1.44 (0.91 to 2.27)	

Cruciferous vegetables (Q4 vs. Q1)	Breast cancer-specific mortality	1.02 (0.80 to 1.30)
Fruit (Q4 vs. Q1)		1.38 (0.88 to 2.17)
Total fat (Q5 vs. Q1)		0.92 (0.53 to 1.60)
Saturated fat (Q5 vs. Q1)		1.55 (0.88 to 2.75)
Trans fat (Q5 vs. Q1)		1.42 (0.80 to 2.52)
Monounsaturated fat (Q5 vs. Q1)		0.89 (0.49 to 1.60)
Polyunsaturated fat (Q5 vs. Q1)		0.90 (0.52 to 1.55)
Carbohydrates (Q5 vs. Q1)		0.93 (0.54 to 1.62)
Protein (Q5 vs. Q1)		1.19 (0.66 to 2.14)
Alcohol (Q5 vs. Q1)		1.27 (0.76 to 2.14)
Vitamin A (Q5 vs. Q1)		1.24 (0.68 to 2.24)
alpha-Carotene (Q5 vs. Q1)		0.98 (0.59 to 1.64)
beta-Carotene (Q5 vs. Q1)		1.05 (0.60 to 1.86)
beta-Cryptoxanthin (Q5 vs. Q1)		0.81 (0.45 to 1.45)
Lutein/zeaxanthin (Q5 vs. Q1)		1.16 (0.62 to 2.19)
Lycopene (Q5 vs. Q1)		1.42 (0.80 to 2.50)

Study	Project (follow-up period)	Countries	Diagnostic	Menopausal status	BC stage	Age	Sample size (n)	Exposures	Outcomes	HR/RR (95% CIs)	Covariates
								Fiber (Q5 vs. Q1)		0.75 (0.38 to 1.49)	
								Whole grain (Q5 vs. Q1)		0.83 (0.46 to 1.48)	
								Calcium (Q5 vs. Q1)		0.59 (0.32 to 1.08)	
								Vitamin D (Q5 vs. Q1)		1.02 (0.58 to 1.79)	
								Dairy (Q4 vs. Q1)		0.94 (0.56 to 1.59)	
								Meat (Q4 vs. Q1)		0.89 (0.50 to 1.60)	
								Vegetables (Q4 vs. Q1)		0.96 (0.38 to 2.45)	
								Cruciferous vegetables (Q4 vs. Q1)		0.95 (0.59 to 1.54)	
								Fruit (Q4 vs. Q1)		1.39 (0.64 to 2.99)	
2011 Belle FN	Health, eating, activity and lifestyle (HEAL; median 6.7 years)	United States	Postdiagnostic	Premenopausal + Postmenopausal	I-IIIA	Mean 55.3 years	688 (Total death =106; BC-specific death =83; BC recurrence=82)	Carbohydrates (>212.9 g/day vs. <110.9 g/day)	All-cause mortality	0.99 (0.39 to 2.50)	Fiber, folate intake, tumor stage, treatment, tamoxifen use
								Glycemic index (Q4 vs. Q1)		1.40 (0.78 to 2.50)	Physical activity, tumor stage, treatment, tamoxifen use
								Glycemic load (Q4 vs. Q1)		1.23 (0.46 to 3.31)	Total energy intake, folate intake, fiber intake, tumor stage, treatment,

Study	Project (follow-up period)	Countries	Diagnostic	Menopausal status	BC stage	Age	Sample size (n)	Exposures	Outcomes	HR/RR (95% CIs)	Covariates
											tamoxifen use
								Fiber (>18.3 g/day vs. <8.8 g/day)		0.53 (0.23 to 1.23)	Total energy intake, folate intake, tumor stage, treatment, tamoxifen use
								Carbohydrates (>212.9 g/day vs. <110.9 g/day)		0.76 (0.27 to 2.17)	Fiber, folate intake, tumor stage, treatment, tamoxifen use
								Glycemic index (Q4 vs. Q1)	Breast cancer-specific mortality	1.60 (0.80 to 3.21)	Physical activity, tumor stage, treatment, tamoxifen use
								Glycemic load (Q4 vs. Q1)		1.11 (0.37 to 3.34)	Total energy intake, folate intake, fiber intake, tumor stage, treatment, tamoxifen use

Study	Project (follow-up period)	Countries	Diagnostic	Menopausal status	BC stage	Age	Sample size (n)	Exposures	Outcomes	HR/RR (95% CIs)	Covariates
								Fiber (>18.3 g/day vs. <8.8 g/day)		0.68 (0.27 to 1.70)	Total energy intake, folate intake, tumor stage, treatment, tamoxifen use
								Carbohydrates (>212.9 g/day vs. <110.9 g/day)	Breast cancer recurrence	0.77 (0.27 to 2.19)	Fiber, folate intake, tumor stage, treatment, tamoxifen use

Study	Project (follow-up period)	Countries	Diagnostic	Menopausal status	BC stage	Age	Sample size (n)	Exposures	Outcomes	HR/RR (95% CIs)	Covariates
								Glycemic index (Q4 vs. Q1)		1.56 (0.77 to 3.13)	Physical activity, tumor stage, treatment, tamoxifen use
								Glycemic load (Q4 vs. Q1)		1.14 (0.38 to 3.44)	Total energy intake, folate intake, fiber intake, tumor stage, treatment, tamoxifen use
								Fiber (>18.3 g/day vs. <8.8 g/day)		0.68 (0.27 to 1.70)	Total energy intake, folate intake, tumor stage, treatment, tamoxifen use
2011 Caan BJ	Women's health eating and living (WHEL; median 7.3 years)	United States	Postdiagnostic	Premenopausal + Postmenopausal	I-III	18-70 years	2,736 (Total death =271; BC recurrence =448)	Total isoflavone (Level 4 vs. Levle1)	All-cause mortality	0.46 (0.20 to 1.05)	Stage, grade, ER/PR status, menopausal status, chemotherapy treatment, radiation, age, education, race, soy supplements, intervention group, presence of hot flash symptoms and their interaction, tamoxifen use
								Total isoflavone (Level 4 vs. Levle1)	Breast cancer recurrence	078 (0.46 to 1.31)	

Study	Project (follow-up period)	Countries	Diagnostic	Menopausal status	BC stage	Age	Sample size (n)	Exposures	Outcomes	HR/RR (95% CIs)	Covariates
2011 George SM	Health, eating, activity and	United States	Postdiagnostic	Premenopausal + Postmenopausal	I-III	Mean 57.9 years	670 (Total death =62; BC-specific death =24)	HEI-2005 (Q4 vs. Q1)	All-cause mortality	0.40 (0.17 to 0.94)	Energy intake, physical activity,
	lifestyle (HEAL; mean 6 years)							HEI-2005 (Q4 vs. Q1)	Breast cancer-specific mortality	0.12 (0.02 to 0.99)	race, stage, tamoxifen use, BMI
2011 Kim EHJ	Nurses' health study (NHS; 1978-1998 through 2004)	United States	Postdiagnostic	Premenopausal + Postmenopausal	I-III	30 to 55 years	2,729 (Total death =572; BC-specific death =302)	AHEI (Q5 vs. Q1)	All-cause mortality	0.85 (0.63 to 1.17)	Time since diagnosis, age, alcohol intake, energy, multivitamins, BMI, weight change, oral contraceptive use, smoking status, physical activity, stage, categories of treatment, age at first birth and parity, menopausal status, postmenopausal hormone use
								DQIR (Q5 vs. Q1)		0.78 (0.58 to 1.07)	
								RFS (Q5 vs. Q1)		1.03 (0.74 to 1.42)	
								aMED (Q5 vs. Q1)		0.87 (0.64 to 1.17)	
								AHEI (Q5 vs. Q1)	Breast cancer-specific mortality	1.53 (0.98 to 2.39)	
								DQIR (Q5 vs. Q1)		0.81 (0.53 to 1.24)	
								RFS (Q5 vs. Q1)		1.54 (0.95 to 2.47)	
								aMED (Q5 vs. Q1)		1.15 (0.74 to 1.77)	
2011 Kwan ML	Life after cancer	United States	Postdiagnostic	Premenopausal +	Invasive BC	Mean 58 years	2,236 (Total	Multivitamins supplements (Yes vs. No)	All-cause mortality	0.92 (0.71 to 1.19)	Age at diagnosis, race/ethnicity,

				Postmenopausal			death =396;				
**Study**	**Project (follow-up period)**	**Countries**	**Diagnostic**	**Menopausal status**	**BC stage**	**Age**	**Sample size (n)**	**Exposures**	**Outcomes**	**HR/RR (95% CIs)**	**Covariates**
								Multivitamins supplements (Yes vs. No)	Breast cancer-specific mortality	0.87 (0.60 to 1.24)	
								Multivitamins supplements (Yes vs. No)	Breast cancer recurrence	0.92 (0.71 to 1.20)	
	epidemiology (LACE; 8.33 years)		Prediagnostic + Postdiagnostic				BC-specific death =212; BC recurrence =380)	Multivitamins supplements (Persistent use vs. Never use)	All-cause mortality	0.79 (0.56 to 1.12)	education, positive nodes, stage, hormone Receptor status, treatment, pre-diagnosis BMI, other antioxidant use, smoking, non-sedentary physical activity, fruit and vegetable consumption
								Fruit and vegetable consumption (Persistent use vs. Never use)	All-cause mortality	0.28 (0.11 to 0.72)	
								Multivitamins supplements (Persistent use vs. Never use)	Breast cancer-specific mortality	0.70 (0.44 to 1.11)	
								Fruit and vegetable consumption (Persistent use vs. Never use)	Breast cancer-specific mortality	0.48 (0.11 to 2.03)	
								Multivitamins supplements (Persistent use vs. Never use)	Breast cancer recurrence	0.76 (0.54 to 1.06)	
								Fruit and vegetable consumption	Breast cancer recurrence	0.58 (0.25 to 1.36)	

								(Persistent use vs. Never use)			
2011 Nechuta S	Shanghai breast cancer survival study (SBCSS; mean 4.1	China	Postdiagnostic	-	Invasive BC	20 to 75 years	4,877 (Total death =444; BC-specific death =389)	Multivitamins supplements (Postdiagnosis use vs. Never postdiagnosis use)	All-cause mortality	0.82 (0.57 to 1.17)	ER/PR status, TNM stage, chemotherapy, radiotherapy,
Study	Project (follow-up period)	Countries	Diagnostic	Menopausal status	BC stage	Age	Sample size (n)	Exposures	Outcomes	HR/RR (95% CIs)	Covariates
	years)							Vitamin E supplements (Postdiagnosis use vs. Never postdiagnosis use)		0.71 (0.46 to 1.11)	Tamoxifen use, education, income, BMI, regular tea consumption, regular exercise participation, daily cruciferous vegetable intake, daily soy protein intake, other vitamin variables
								Vitamin C supplements (Postdiagnosis use vs. Never postdiagnosis use)		0.81 (0.61 to 1.07)	
								Antioxidant supplements (Postdiagnosis use vs. Never postdiagnosis use)		0.82 (0.65 to 1.02)	
							4,325 (BC recurrence=532)	Multivitamins supplements (Postdiagnosis use vs. Never postdiagnosis use)	Breast cancer recurrence	0.74 (0.53 to 1.03)	

Study	Project (follow-up period)	Countries	Diagnostic	Menopausal status	BC stage	Age	Sample size (n)	Exposures	Outcomes	HR/RR (95% CIs)	Covariates
								Vitamin E supplements (Postdiagnosis use vs. Never postdiagnosis use)		0.65 (0.43 to 0.97)	
								Vitamin C supplements (Postdiagnosis use vs. Never postdiagnosis use)		0.81 (0.63 to 1.03)	
								Antioxidant supplements (Postdiagnosis use vs. Never postdiagnosis use)		0.78 (0.61 to 0.95)	
2011 Saquib J	Women's healthy eating and living (WHEL; median 9.0 years)	United States	Postdiagnostic	-	I-IIIA	Mean 53 years	2,939 (Total death =388)	Retinol supplements (Upper limit for each micronutrient vs. Adequate micronutrients intake)	All-cause mortality	0.90 (0.61 to 1.48)	Age at randomization, tumor stage, tumor grade, time since diagnosis, BMI, smoking, randomization group, hot flashes
								Niacin supplements (Upper limit for each micronutrient vs. Adequate		1.02 (0.83 to 1.26)	

								micronutrients intake)			
								Vitamin B6 supplements (Upper limit for each micronutrient vs. Adequate micronutrients intake)		0.90 (0.63 to 1.53)	
								Folate supplements (Upper limit for each micronutrient vs. Adequate micronutrients intake)		1.30 (0.89 to 1.78)	
								Vitamin C supplements (Upper limit for each micronutrient vs. Adequate micronutrients intake)		1.10 (0.79 to 1.60)	

Study	Project (follow-up period)	Countries	Diagnostic	Menopausal status	BC stage	Age	Sample size (n)	Exposures	Outcomes	HR/RR (95% CIs)	Covariates
								Vitamin E supplements (Upper limit for each micronutrient vs. Adequate		1.60 (0.73 to 3.71)	

		micronutrients intake)	
		Vitamin D supplements (Upper limit for each micronutrient vs. Adequate micronutrients intake)	0.90 (0.13 to 7.11)
		Calcium supplements (Upper limit for each micronutrient vs. Adequate micronutrients intake)	1.10 (0.68 to 1.71)
		Iron supplements (Upper limit for each micronutrient vs. Adequate micronutrients intake)	1.30 (0.93 to 1.97)
		Magnesium supplements (Upper limit for each micronutrient vs. Adequate micronutrients intake)	1.03 (0.69 to 1.53)

Study	Project (follow-up period)	Countries	Diagnostic	Menopausal status	BC stage	Age	Sample size (n)	Exposures	Outcomes	HR/RR (95% CIs)	Covariates
								Selenium supplements (Upper limit for each micronutrient vs. Adequate micronutrients intake)		1.50 (0.78 to 2.96)	
								Zinc supplements (Upper limit for each micronutrient vs. Adequate micronutrients intake)		1.00 (0.68 to 1.48)	
2012 Greenlee H	Life after cancer epidemiology (LACE; mean 8.3 years)	United States	Postdiagnostic	-	I-IIIA	Mean 58.3 years	2,264 (Total death =393; BC-specific death =214; BC recurrence =375)	Multivitamins supplements (Frequent use vs. No use)	All-cause mortality	0.84 (0.65 to 1.08)	Age at diagnosis, race, education, breast cancer stage at diagnosis, number positive lymph nodes, tumor hormone receptor status, chemotherapy, radiation therapy, BMI, smoking, alcohol consumption, physical activity,
								Vitamin C supplements (Frequent use vs. No use)		0.78 (0.61 to 1.01)	
								Vitamin E supplements (Frequent use vs. No use)		0.75 (0.59 to 0.96)	
								Carotenoids supplements (Frequent use vs. No use)		1.63 (1.06 to 2.50)	

Study	Project (follow-up period)	Countries	Diagnostic	Menopausal status	BC stage	Age	Sample size (n)	Exposures	Outcomes	HR/RR (95% CIs)	Covariates
								beta-Carotene supplements (Frequent use vs. No use)		1.18 (0.71 to 1.97)	
								Lycopene supplements (Frequent use vs. No use)		1.38 (0.41 to 4.61)	
								Selenium supplements (Frequent use vs. No use)		0.80 (0.45 to 1.41)	
								Zinc supplements (Frequent use vs. No use)		0.75 (0.46 to 1.21)	
								Multivitamins supplements (Frequent use vs. No use)	Breast cancer-specific mortality	0.79 (0.56 to 1.12)	daily servings of fruits and vegetable, comorbidity score
								Vitamin C supplements (Frequent use vs. No use)		0.82 (0.58 to 1.16)	
								Vitamin E supplements (Frequent use vs. No use)		0.85 (0.61 to 1.18)	
								Carotenoids supplements (Frequent use vs. No use)		1.93 (1.14 to 3.28)	

								beta-Carotene supplements (Frequent use vs. No use)		1.33 (0.69 to 2.55)	
								Lycopene supplements (Frequent use vs. No use)		2.09 (0.59 to 7.43)	
								Selenium supplements (Frequent use		0.90 (0.45 to 1.79)	
								Zinc supplements (Frequent use vs. No use)		0.82 (0.44 to 1.53)	
								Multivitamins supplements (Frequent use vs. No use)	Breast cancer recurrence	0.82 (0.63 to 1.06)	

Study	Project (follow-up period)	Countries	Diagnostic	Menopausal status	BC stage	Age	Sample size (n)	Exposures	Outcomes	HR/RR (95% CIs)	Covariates
								Vitamin C supplements (Frequent use vs. No use)		0.71 (0.54 to 0.92)	
								Vitamin E supplements (Frequent use vs. No use)		0.70 (0.54 to 0.90)	
								Carotenoids supplements (Frequent use vs. No use)		1.23 (0.76 to 1.96)	
								beta-Carotene supplements		0.89 (0.50 to 1.60)	

								(Frequent use vs. No use)			
								Lycopene supplements (Frequent use vs. No use)		1.17 (0.35 to 3.89)	
								Selenium supplements (Frequent use		0.89 (0.53 to 1.49)	
								Zinc supplements (Frequent use vs. No use)		0.79 (0.49 to 1.28)	
2012 Harris HR(a)	Swedish mammography cohort (SMC; mean 8.8 years)	Sweden	Prediagnostic	Premenopausal + Postmenopausal	Invasive BC	Mean 65.5 years	3,234 (total death =973; BC-specific mortality =394)	Coffee (>4 cups/day vs. <1 cup/day)	All-cause mortality	1.12 (0.84 to 1.51)	Age, energy intake, education level, marital status, menopausal status at diagnosis, BMI, alcohol intake,
								Tea (>2 cups/day vs. 0 cup/day)		0.94 (0.72 to 1.23)	
								Coffee (>4 cups/day vs. <1 cup/day)	Brest cancer-specific mortality	1.14 (0.71 to 1.83)	
								Tea (>2 cups/day vs. 0 cup/day)		1.02 (0.67 to 1.55)	
Study	Project (follow-up period)	Countries	Diagnostic	Menopausal status	BC stage	Age	Sample size (n)	Exposures	Outcomes	HR/RR (95% CIs)	Covariates
											calendar year of diagnosis, disease stage, grade, radiation treatment, chemotherapy, hormonal treatment

2012 Harris HR(b)	Swedish mammography cohort (SMC; mean 8.2 years)	Sweden	Prediagnostic	Premenopausal + Postmenopausal	Invasive BC	mean 64.3 years	3,146 (Total death =860; BC-specific death =385)	Alcohol intake (>10 g/day vs. Non-drinker)	All-cause mortality	1.03 (0.71 to 1.51)	Age, energy intake, education level, marital status, menopausal status at diagnosis, BMI, calendar year of diagnosis, disease stage, grade, radiation treatment, chemotherapy and/or hormonal treatment
								Alcohol intake (>10 g/day vs. Non-drinker)	Breast cancer-specific mortality	1.36 (0.82 to 2.26)	
2012 Harris HR(c)	Swedish mammography cohort	Sweden	Prediagnostic	Premenopausal + Postmenopausal	Invasive BC	Mean 65.1 years	3,116 (Total death =852;	Folate intake (>246 µg/day vs. <190 µg/day)	All-cause mortality	0.78 (0.65 to 0.94)	Age, energy intake, education
Study	Project (follow-up period)	Countries	Diagnostic	Menopausal status	BC stage	Age	Sample size (n)	Exposures	Outcomes	HR/RR (95% CIs)	Covariates
	(SMC; mean 8.3 years)						BC-specific death =381)	Folate intake (>246 µg/day vs. <190 µg/day)	Breast cancer-specific mortality	0.78 (0.58 to 1.03)	level, marital status, menopausal status at diagnosis, BMI, alcohol intake, calendar year of diagnosis, disease stage, grade, radiation treatment, chemotherapy, hormonal treatment

Study	Project (follow-up period)	Countries	Diagnostic	Menopausal status	BC stage	Age	Sample size (n)	Exposures	Outcomes	HR/RR (95% CIs)	Covariates
2012 Nechuta SJ	After breast cancer pooling project (ABCPP; mean 7.4 years)	United States and China	Postdiagnostic	Premenopausal + Postmenopausal	I-III	Mean 54.5 years	9,514 (Total death =1,171; BC-specific death =881; BC recurrence =1,348)	Isoflavone (T3 vs. T1)	All-cause mortality	0.87 (0.70 to 1.10)	Age at diagnosis, ER/PR status, TNM stage, chemotherapy, radiotherapy, hormonal therapy, smoking, BMI, exercise, cruciferous vegetable intake, parity, menopausal status, study, race, education
								Isoflavone (T3 vs. T1)	Breast cancer-specific mortality	0.83 (0.64 to 1.07)	
								Isoflavone (T3 vs. T1)	Breast cancer recurrence	0.75 (0.61 to 0.92)	
2012 Vrieling A	Mamma carcinoma risk factor investigation (MARIE; median 5.5 years)	Germany	Prediagnostic	Postmenopausal	I-IIIA	Mean 62.7 years	2,522 (Total death =316; BC-specific death =235; BC recurrence =247)	Total alcohol intake (>12.0 g/day vs. <0.5 g/day)	All-cause mortality	1.28 (0.90 to 1.81)	Age at diagnosis, study center, tumor size, nodal status, metastases, tumor grade, ERPR status, radiotherapy, HRT use at diagnosis, mode of detection
								Total alcohol intake (>12.0 g/day vs. <0.5 g/day)	Breast cancer-specific mortality	1.74 (1.13 to 2.67)	
								Total alcohol intake (>12.0 g/day vs. <0.5 g/day)	Breast cancer recurrence	1.08 (0.73 to 1.58)	
2013 Conroy SM	Multiethnic cohort (MEC; mean 6.2 years)	United States	Prediagnostic	Postmenopausal	Invasive BC	Mean 68.6 years	3,842 (Total death =804; BC-specific	Soy products (T3 vs. T1)	All-cause mortality	1.03 (0.81 to 1.33)	BMI, age at diagnosis, ethnicity, energy intake, stage, hormone receptor status, treatment,
								Dietary isoflavones (T3 vs. T1)		0.99 (0.82 to 1.20)	
								Soy products		1.03	

Study	Project (follow-up period)	Countries	Diagnostic	Menopausal status	BC stage	Age	Sample size (n)	Exposures	Outcomes	HR/RR (95% CIs)	Covariates
							death =376)	(T3 vs. T1)		(0.71 to 1.50)	cardiovascular comorbidity, history of
								Dietary isoflavones (T3 vs. T1)	Breast cancer-specific mortality	0.95 (0.71 to 1.28)	
											diabetes, smoking status, years between cohort entry and diagnosis
2013 Harris HR	Swedish mammography cohort (SMC; median 7.8 years)	Sweden	Prediagnostic	Premenopausal + Postmenopausal	Invasive BC	Mean 65 years	3,405 (Total death =1,055; BC-specific death =416)	Vitamin C (Q4 vs. Q1)	All-cause mortality	0.84 (0.71 to 1.00)	Age, energy intake, education level, marital status, menopausal status at diagnosis, BMI, alcohol intake, calendar year of diagnosis, disease stage, grade, radiation treatment, chemotherapy, hormonal treatment
								Vitamin C (Q4 vs. Q1)	Breast cancer-specific mortality	0.75 (0.57 to 0.99)	
2013 Holm M	Danish cohort 'diet, cancer and health' (DCH;	Denmark	Prediagnostic	Premenopausal + Postmenopausal	I-III	50 to 64 years	1,052 (BC-specific death =106; BC	Alcohol (>2 units/day vs. <1 unit/day)	Breast cancer-specific mortality	1.10 (0.67 to 1.82)	Tumor size, lymph node status, receptor status
Study	Project (follow-up period)	Countries	Diagnostic	Menopausal status	BC stage	Age	Sample size (n)	Exposures	Outcomes	HR/RR (95% CIs)	Covariates

	median 6.3 years for mortality and median 6 years for recurrence)						recurren ce =110)	Alcohol (>2 units/day vs. <1 unit/day)	Breast cancer recurrence	1.65 (1.02 to 2.67)	and grade, BMI, smoking, menopausal status, HRT use, education level, physical activity, total folate intake
2013 Inoue-Choi M	Iowa women's health study (IWHS; mean 8.6 years)	United States	Prediagnostic + Postdiagnostic	Postmenopausal	Invasive BC	Mean 78.9 years	938 (Total death =203; BC-specific death =75)	WCRF/AICR recommendation adherence score (Q4 vs. Q1)	All-cause mortality	0.61 (0.39 to 0.96)	Age, total number of comorbid conditions, current smoking, cancer stage, cancer type, cancer treatment, subsequent cancer diagnosis before 2004, current cancer treatment, person-years since cancer diagnosis
								WCRF/AICR recommendation adherence score (Q4 vs. Q1)	Breast cancer-specific mortality	0.88 (0.41 to 1.91)	
Study	Project (follow-up period)	Countries	Diagnostic	Menopausal status	BC stage	Age	Sample size (n)	Exposures	Outcomes	HR/RR (95% CIs)	Covariates
2013 Izano MA	Nurses' health study (NHS; median 9.3 years)	United States	Prediagnostic + Postdiagnostic	Premenopausal + Postmenopausal	I-III	Mean 60.4 years	4,103 (Total death =981; BC-specific death =453; BC	DASH (Q5 vs. Q1)	Breast cancer-specific mortality	0.85 (0.61 to 1.19)	Time since diagnosis, age at diagnosis, energy intake, BMI, BMI change, age at first birth and parity, oral contraceptive
								AHEI-2010 (Q5 vs. Q1)		1.07 (0.77 to 1.49)	

							recurrence=38)				use, menopausal status, HRT use, smoking, stage of disease, radiation treatment, chemotherapy, hormonal treatment, physical activity
2013 Kroenke CH	Life after cancer epidemiology (LACE; median 11.8 years)	United States	Prediagnostic + Postdiagnostic	Premenopausal + Postmenopausal	I-IIIA	Mean 58.6 years	1,893 (Total death =372; BC-specific death =189; BC recurrence	Total dairy intake (>2.0 servings/day vs. 0-1.0 serving/day)	All-cause mortality	1.39 (1.02 to 1.90)	Age at diagnosis, time between diagnosis and dietary assessment, age, race, education
Study	Project (follow-up period)	Countries	Diagnostic	Menopausal status	BC stage	Age	Sample size (n)	Exposures	Outcomes	HR/RR (95% CIs)	Covariates
							=349)	Total dairy intake (>2.0 servings/day vs. 0-1.0 serving/day)	Breast cancer-specific mortality	1.26 (0.81 to 1.95)	, cancer stage at diagnosis, tumor size, human epidermal growth receptor 2
								Total dairy intake (>2.0 servings/day vs. 0-1.0 serving/day	Breast cancer recurrence	1.13 (0.83 to 1.54)	neu status, nodal status, estrogen receptor status, chemotherapy, radiation, tamoxifen, comorbidity, menopausal status, smoking status, BMI,

											energy intake, alcohol intake, red meat intake, fiber intake, fruit intake
2013 Kwan ML	After breast cancer	United States	Postdiagnostic	Premenopausal + Postmenopausal	I-III	Mean 58.8 years	9,325 (Total death	Alcohol intake (Regular high vs. Non-drinker)	All-cause mortality	0.79 (0.63 to 1.00)	Age at diagnosis, AJCC
Study	Project (follow-up period)	Countries	Diagnostic	Menopausal status	BC stage	Age	Sample size (n)	Exposures	Outcomes	HR/RR (95% CIs)	Covariates
	pooling project (ABCPP; mean 10.3 years)						=1,542)				stage, race/ethnicity, education, menopausal status around diagnosis, hormone receptor status, surgery, treatment, smoking, physical activity, pre-diagnosis BMI, comorbidity
							9,306 (BC-specific death =911)	Alcohol intake (Regular high vs. Non-drinker)	Breast cancer-specific mortality	0.80 (0.59 to 1.09)	
							9,151 (BC recurrence =1,487)	Alcohol intake (Regular high vs. Non-drinker)	Breast cancer recurrence	1.04 (0.84 to 1.31)	
2013 Newcomb PA	Collaborative women's longevity study (CWLS; median 11.3 years)	United States	Prediagnostic	Premenopausal + Postmenopausal	-	20 to 79 years	22,890 (Total death =7,780; BC-specific death =19,220)	Alcohol consumption (>10 drinks/week vs. 0 drink/week)	All-cause mortality	0.96 (0.88 to 1.05)	Age at diagnosis, stage of disease at diagnosis, state of residence at diagnosis, study phase, family history of breast cancer, age at first birth,
								Wine consumption (>7 drinks/week vs. 0 drink/week)		0.97 0.85 to 1.09	

								Beer consumption (>7 drinks/week vs. 0 drink/week)		1.13 (1.01 to 1.26)	menopausal status,
								Spirits consumption (>7 drinks/week vs. 0 drink/week)		1.05 (0.95 to 1.15)	

Study	Project (follow-up period)	Countries	Diagnostic	Menopausal status	BC stage	Age	Sample size (n)	Exposures	Outcomes	HR/RR (95% CIs)	Covariates
								Alcohol consumption (>10 drinks/week vs. 0 drink/week)	Breast cancer-specific mortality	0.89 (0.77 to 1.04)	hormone therapy use, BMI, smoking status, education, mammography
								Wine consumption (>7 drinks/week vs. 0 drink/week)		1.11 (0.93 to 1.34)	
								Beer consumption (>7 drinks/week vs. 0 drink/week)		0.96 (0.81 to 1.14)	
								Spirits consumption (>7 drinks/week vs. 0 drink/week)		0.86 (0.73 to 1.02)	
			Postdiagnostic				4,881 (Total death	Alcohol consumption (>10	All-cause mortality	0.64 (0.47 to 0.88)	

Study	Project (follow-up period)	Countries	Diagnostic	Menopausal status	BC stage	Age	Sample size (n)	Exposures	Outcomes	HR/RR (95% CIs)	Covariates
							=1,069; BC-specific death =276)	drinks/week vs. 0 drink/week)			
								Wine consumption (>7 drinks/week vs. 0 drink/week)		0.73 (0.51 to 1.05)	
								Beer consumption (>7 drinks/week vs. 0 drink/week)		0.91 (0.53 to 1.57)	
								Spirits consumption (>7 drinks/week vs. 0 drink/week)		0.69 (0.49 to 0.98)	
								Alcohol consumption (>10 drinks/week vs. 0 drink/week)	Breast cancer-specific mortality	0.83 (0.45 to 1.54)	
								Wine consumption (>7 drinks/week vs. 0 drink/week)		1.45 (0.77 to 2.73)	
								Beer consumption (>7 drinks/week vs. 0 drink/week)		0.94 (0.37 to 2.39)	

								Spirits consumption (>7 drinks/week vs. 0 drink/week)		0.83 (0.43 to 1.62)	
2013 Poole EM	After breast cancer pooling project (ABCPP; -)	United States and China	Postdiagnostic	-	I-III	Mean 56.8 years	12,019 (Total death =1,298; BC-specific death =849)	Multivitamin supplements (Yes vs. No)	All-cause mortality	0.94 (0.83 to 1.07)	Age at diagnosis, exercise, stage, treatment, BMI, menopausal status, smoking, use of all supplements
								Vitamin A supplements (Yes vs. No)		1.06 (0.82 to 1.36)	
								Vitamin B supplements (Yes vs. No)		0.96 (0.81 to 1.15)	
								Vitamin C supplements (Yes vs. No)		0.87 (0.76 to 1.01)	
								Vitamin D supplements (Yes vs. No)		0.95 (0.72 to 1.24)	
								Vitamin E supplements (Yes vs. No)		0.92 (0.79 to 1.07)	
								Antioxidants supplements (Yes vs. No)		0.84 (0.72 to 0.99)	
								Multivitamin supplements (Yes vs. No)	Breast cancer-specific mortality	0.95 (0.82 to 1.11)	
								Vitamin A supplements (Yes vs. No)		0.95 (0.68 to 1.34)	
								Vitamin B supplements (Yes vs. No)		0.98 (0.80 to 1.21)	

								Vitamin C supplements		0.94 (0.79 to 1.12)	
Study	Project (follow-up period)	Countries	Diagnostic	Menopausal status	BC stage	Age	Sample size (n)	Exposures	Outcomes	HR/RR (95% CIs)	Covariates
								(Yes vs. No)			
								Vitamin D supplements (Yes vs. No)		0.97 (0.68 to 1.38)	
								Vitamin E supplements (Yes vs. No)		0.89 (0.72 to 1.10)	
								Antioxidants supplements (Yes vs. No)		0.88 (0.74 to 1.03)	
							11,672 (BC recurrence =1,325)	Multivitamin supplements (Yes vs. No)	Breast cancer recurrence	0.97 (0.86 to 1.09)	
								Vitamin A supplements (Yes vs. No)		1.16 (0.80 to 1.70)	
								Vitamin B supplements (Yes vs. No)		0.94 (0.79 to 1.11)	
								Vitamin C supplements (Yes vs. No)		0.98 (0.85 to 1.12)	
								Vitamin D supplements (Yes vs. No)		0.92 (0.62 to 1.35)	
								Vitamin E supplements (Yes vs. No)		0.90 (0.78 to 1.03)	
								Antioxidants supplements (Yes vs. No)		0.94 (0.83 to 1.07)	

Study	Project (follow-up period)	Countries	Diagnostic	Menopausal status	BC stage	Age	Sample size (n)	Exposures	Outcomes	HR/RR (95% CIs)	Covariates
2013 Vrieling A	Mamma carcinoma risk factor investigation (MARIE; median 5.5	Germany	Prediagnostic	Postmenopausal	Invasive BC	50 to 74 years	2,184 (Total death =316; BC-specific death =235; BC	Healthy dietary pattern (Q4 vs. Q1)	All-cause mortality	0.87 (0.61 to 1.23)	Age at diagnosis, study center, tumor size, nodal status, metastas
	years)						recurrence=247)	Unhealthy dietary pattern (Q4 vs. Q1)		1.34 (0.93 to 1.94)	es, tumour grade, ERPR status, radiotherapy, HRT use at diagnosis, mode of detection, total energy intake
								Healthy dietary pattern (Q4 vs. Q1)	Breast cancer-specific mortality	0.89 (0.59 to 1.35)	
								Unhealthy dietary pattern (Q4 vs. Q1)		0.99 (0.64 to 1.52)	
								Healthy dietary pattern (Q4 vs. Q1)	Breast cancer recurrence	0.71 (0.46 to 1.06)	
								Unhealthy dietary pattern (Q4 vs. Q1)		0.91 (0.61 to 1.36)	
2014 Geroge SM	Women's health initiative (WHI-observational studies; median 9.6 years)	United States	Prediagnostic + Postdiagnostic	Postmenopausal	Invasive BC	Mean 63.6 years	2,317 (Total death =415; BC-specific death =188)	HEI-2005 (Q4 vs. Q1)	All-cause mortality	0.74 (0.55 to 0.99)	Age, WHI component, ethnicity, income, education, stage, ER status, PR status, time since diagnosis, energy intake in kcals, physical activity, serving of alcohol per week, use of
								HEI-2005 (Q4 vs. Q1)	Breast cancer-specific mortality	0.91 (0.60 to 1.40)	

											postmenopausal hormone therapy
Study	Project (follow-up period)	Countries	Diagnostic	Menopausal status	BC stage	Age	Sample size (n)	Exposures	Outcomes	HR/RR (95% CIs)	Covariates
2015 Bao PP	Shanghai breast cancer survival study (SBCSS; median 9.1 years)	China	Postdiagnostic	Premenopausal + Postmenopausal	Triple-negative BC	Mean 53.4 years	518 (Total death =128; BC-specific death =235; BC recurrence=247)	Tea consumption (first 6 months post-diagnosis; Yes vs. No)	All-cause mortality	0.58 (0.29 to 1.16)	Age at diagnosis, education, marital status, Chalson comorbidity index, menopausal status, BMI at baseline, exercise participation at baseline, soy protein intake, chemotherapy, radiotherapy, TNM stage
								Tea consumption (first 18 months post-diagnosis; Yes vs. No)		0.76 (0.47 to 1.21)	
								Tea consumption (first 36 months post-diagnosis; Yes vs. No)		0.47 (0.29 to 0.79)	
								Tea consumption (first 60 months post-diagnosis; Yes vs. No)		0.57 (0.34 to 0.93)	
								Tea consumption (first 6 months post-diagnosis; Yes vs. No)	Breast cancer-specific mortality or recurrence	0.60 (0.29 to 1.27)	
								Tea consumption (first 18 months post-diagnosis; Yes vs. No)		0.58 (0.33 to 1.02)	

								Tea consumption (first 36 months post-diagnosis; Yes vs. No)		0.46 (0.26 to 0.83)	
								Tea consumption (first 60 months post-diagnosis; Yes vs. No)		0.54 (0.31 to 0.96)	
2015 Jeffreys M	UK Clinical practice research datalink (CPRD; median 2.5	UK	Prediagnostic	Postmenopausal	-	-	11,112 (Total death =2,103)	Vitamin D supplements (Any prescription vs. None)	All-cause mortality	0.78 (0.70 to 0.88)	Age, period, smoking, alcohol, BMI, area-level deprivation
Study	Project (follow-up period)	Countries	Diagnostic	Menopausal status	BC stage	Age	Sample size (n)	Exposures	Outcomes	HR/RR (95% CIs)	Covariates
	years)										
2015 Kyro C	European prospective investigation into cancer and nutrition (EPIC; median 6.3 years)	Europe	Prediagnostic	Premenopausal	In situ and metastatic BC	Median 59 years	2,804 (Total death =295; BC-specific death =186)	Flavonoids	All-cause mortality	0.94 (0.80 to 1.10)	Age, country, alcohol, BMI, HRT use, schooling, smoking, physical activity, intake of other polyphenol classes, ER receptor status, cancer stage, grading of tumor
								Phenolic acids		1.04 (0.90 to 1.20)	
								Stilbenes		0.99 (0.92 to 1.06)	
								Lignans		1.26 (1.05 to 1.51)	
								Flavonoids	Breast cancer-specific mortality	1.02 (0.83 to 1.26)	
								Phenolic acids		1.00 (0.83 to 1.21)	
								Stilbenes		0.99 (0.91 to 1.08)	
								Lignans		1.24 (0.98 to 1.58)	
								Flavonoids	All-cause mortality	1.02	

										(0.95 to 1.10)	
								Phenolic acids		0.97 (0.91 to 1.05)	
								Stilbenes		0.97 (0.95 to 1.00)	
				Postmenopausal			8,978 (Total death =1,187; BC-specific death =567)	Lignans		0.94 (0.86 to 1.04)	
								Flavonoids	Breast cancer-specific mortality	1.04 (0.93 to 1.15)	
								Phenolic acids		0.99 (0.89 to 1.10)	
								Stilbenes		0.97 (0.94 to 1.00)	
								Lignans		0.83 (0.72 to 0.96)	
2016 McCullough ML	ACS's cancer prevention study-II nutrition cohort	United States	Prediagnostic	-	-	Mean 70.7 years	4,452 (Total death =1,204; BC-specific death	ACS guidelines diet score (3-5 vs. 0-2)	All-cause mortality	1.01 (0.88 to 1.17)	Age at diagnosis, diagnosis year, tumor stage at diagnosis,

Study	Project (follow-up period)	Countries	Diagnostic	Menopausal status	BC stage	Age	Sample size (n)	Exposures	Outcomes	HR/RR (95% CIs)	Covariates
	(CPS-II Nutrition Cohort; mean 9.8 years)						=398)	ACS guidelines diet score (6-9 vs. 0-2)		1.00 (0.84 to 1.18)	Tumor grade at diagnosis, ER status, PR status, surgery, chemotherapy, radiation, hormone therapy, aromatase inhibitor uses
								Fruit and vegetable intake (Q3 vs. Q1)		1.06 (0.85 to 1.33)	
								Total grain intake (Q3 vs. Q1)		1.08 (0.91 to 1.29)	

Study	Project (follow-up period)	Countries	Diagnostic	Menopausal status	BC stage	Age	Sample size (n)	Exposures	Outcomes	HR/RR (95% CIs)	Covariates
								Red and processed meat intake (Q3 vs. Q1)		0.88 (0.73 to 1.06)	and/or herceptin use, BMI, smoking, physical activity, energy intake
								ACS guidelines diet score (3-5 vs. 0-2)	Breast cancer-specific mortality	1.00 (0.78 to 1.29)	
								ACS guidelines diet score (6-9 vs. 0-2)		1.06 (0.79 to 1.42)	
								Fruit and vegetable intake (Q3 vs. Q1)		1.00 (0.66 to 1.50)	
								Total grain intake (Q3 vs. Q1)		1.07 (0.79 to 1.46)	
								Red and processed meat intake (Q3 vs. Q1)		1.10 (0.80 to 1.52)	
			Postdiagnostic				2,152 (Total death =640; BC-specific death =192)	ACS guidelines diet score (3-5 vs. 0-2)	All-cause mortality	1.00 (0.81 to 1.23)	
								ACS guidelines diet score (6-9 vs. 0-2)		0.93 (0.73 to 1.18)	
								Fruit and vegetable intake (Q3 vs. Q1)		1.03 (0.80 to 1.33)	
								Total grain intake (Q3 vs. Q1)		1.09 (0.86 to 1.38)	

								Red and processed meat intake (Q3 vs. Q1)		0.64 (0.49 to 0.84)	
								ACS guidelines diet score (3-5 vs. 0-2)	Breast cancer-specific mortality	1.45 (0.95 to 2.20)	
								ACS guidelines diet score (6-9 vs. 0-2)		1.44 (0.90 to 2.30)	
								Fruit and vegetable intake (Q3 vs. Q1)		1.31 (0.83 to 2.06)	
								Total grain intake (Q3 vs. Q1)		1.24 (0.81 to 1.88)	
								Red and processed meat intake (Q3 vs. Q1)		0.88 (0.54 to 1.43)	
2016 Neuhouser ML	Health, eating activity and lifestyle study (HEAL; median 4.5 years)	United States	Postdiagnostic	-	I-IIIA	Mean 57.9 years	707 (Total death =149; BC-specific death =70)	Natural products supplements	All-cause mortality	0.95 (0.67 to 1.35)	Race/ethnicity, BMI, stage of disease, breast cancer treatment, tamoxifen use, Charlson comorbidity score, weekly MET-hours of sport, recreational physical activity
								Natural products supplements	Breast cancer-specific mortality	1.15 (0.69 to 1.94)	

Study	Project (follow-up period)	Countries	Diagnostic	Menopausal status	BC stage	Age	Sample size (n)	Exposures	Outcomes	HR/RR (95% CIs)	Covariates
2016 Tao MH	Western New York exposure and breast cancer (WEB; mean 7.3 years)	United States	Prediagnostic	Premenopausal + Postmenopausal	I-IV	Mean 58.4 years	1,170 (Total death =170; BC-specific death =100)	Dietary calcium intake (>858.13 g/day vs. <558.27 g/day)	All-cause mortality	0.84 (0.49 to 1.45)	age at diagnosis, race, education, BMI, physical activity, menopausal status, TNM, radiotherapy, tamoxifen therapy, ER status, intakes of total energy and total vitamin D, calcium and magnesium intakes
								Dietary magnesium intake (>268.15 g/day vs. <193.64 g/day)		0.50 (0.28 to 0.90)	
								Dietary calcium intake (>858.13 g/day vs. <558.27 g/day)	Breast cancer-specific mortality	1.31 (0.63 to 2.76)	
								Dietary magnesium intake (>268.15 g/day vs. <193.64 g/day)		0.70 (0.33 to 1.49)	
2017 Holmes MD	Nurses' health study (NHS; 1976-2004 through 2010)	United States	Prediagnostic	Premenopausal + Postmenopausal	I-III	30 to 55 years	6,348 (Total death =1,847; BC-specific death =919; BC recurrence =1,046)	Total protein (Q5 vs. Q1)	Breast cancer recurrence	0.84 (0.69 to 1.03)	age at diagnosis, time since diagnosis, energy intake, BMI, weight change, age at first birth and parity, oral contraceptive use,
								Animal protein (Q5 vs. Q1)		0.74 (0.61 to 0.91)	
								Vegetable protein (Q5 vs. Q1)		1.29 (1.05 to 1.59)	
								Essential amino acids (Q5 vs. Q1)		0.86 (0.71 to 1.05)	
								Branched-chain amino acids (Q5 vs. Q1)		0.82 (0.68 to 1.00)	
Study	Project (follow-up period)	Countries	Diagnostic	Menopausal status	BC stage	Age	Sample size (n)	Exposures	Outcomes	HR/RR (95% CIs)	Covariates

Study	Project (follow-up period)	Countries	Diagnostic	Menopausal status	BC stage	Age	Sample size (n)	Exposures	Outcomes	HR/RR (95% CIs)	Covariates
								Red meat (Q5 vs. Q1)		1.03 (0.83 to 1.29)	menopausal status and hormone therapy use, aspirin use, alcohol, smoking, physical activity, tumor stage, radiation treatment, other treatment, calendar year
								Poultry (Q5 vs. Q1)		0.85 (0.69 to 1.05)	
								Fish (Q5 vs. Q1)		0.93 (0.76 to 1.15)	
								High-fat dairy (Q5 vs. Q1)		1.09 (0.88 to 1.35)	
								Low-fat dairy (Q5 vs. Q1)		0.84 (0.69 to 1.04)	
2017 Zeinomar N	The breast cancer family registry (BCFR; mean 9.1 years)	United States	Prediagnostic	Premenopausal + Postmenopausal	Invasive BC	Mean 54.6 years	1,116 (Total death =211; BC-specific death =58)	Alcohol (>3 drinks/week vs. Non-drinkers)	All-cause mortality	1.16 (0.85 to 1.58)	age at baseline, BMI, ethnicity, menopausal status, age at menarche, HRT, and cigarette smoking
								Alcohol (>3 drinks/week vs. Non-drinkers)	Breast cancer-specific mortality	0.96 (0.49 to 1.89)	age at baseline, BMI, ethnicity, physical activity, age at first live birth,
											menopause status, oral contraceptive use, education level, breast feeding duration, and cigarette smoking

Study	Project (follow-up period)	Countries	Diagnostic	Menopausal status	BC stage	Age	Sample size (n)	Exposures	Outcomes	HR/RR (95% CIs)	Covariates
2017 Zhang FF	The breast cancer family registry (BCFR; median 9.4 years)	United States and Canada	Prediagnostic + Postdiagnostic	Premenopausal + Postmenopausal	Invasive BC	Mean 51.8 years	6,235 (Total death =1,224)	Isoflavone (Q4 vs. Q1)	All-cause mortality	0.79 (0.64 to 0.97)	age, study site, total calorie intake, race, education, total fiber intake, HEI-2010, treatment type, recreational physical activity, BMI, alcohol use, smoking status
			Prediagnostic				4,769 (Total death =963)	Isoflavone (Q4 vs. Q1)		0.84 (0.66 to 1.06)	
			Postdiagnostic				1,466 (Total death =261)	Isoflavone (Q4 vs. Q1)		0.65 (0.41 to 1.00)	
2017 Zucchetto A	Italian women cohort (median 12.6 years)	Italy	Prediagnostic	Premenopausal + Postmenopausal	I-IV	-	1,453 (Total death =503; BC-specific death =398)	DII (T3 vs. T1)	All-cause mortality	1.00 (0.78 to 1.28)	Area of residence, calendar year of diagnosis, age at
								DII (T3 vs. T1)	Breast cancer-specific mortality	0.97 (0.73 to 1.27)	diagnosis, education, menopausal status, smoking habit, BMI, total energy intake, hormone receptor status, TNM tumour stage
				Premenopausal			553 (Total death =152; BC-specific death =137)	DII (T3 vs. T1)	All-cause mortality	0.84 (0.52 to 1.34)	
								DII (T3 vs. T1)	Breast cancer-specific mortality	0.76 (0.46 to 1.26)	
				Postmenopausal			900 (Total death =351; BC-specific death =261)	DII (T3 vs. T1)	All-cause mortality	1.05 (0.78 to 1.40)	
								DII (T3 vs. T1)	Breast cancer-specific mortality	1.07 (0.77 to 1.51)	

Study	Project (follow-up period)	Countries	Diagnostic	Menopausal status	BC stage	Age	Sample size (n)	Exposures	Outcomes	HR/RR (95% CIs)	Covariates
2018 Furrer D	The Centre des Maladies du Sein Deschenes-Fabia (CMSDF; median 7.4 years)	Canada	Prediagnostic	-	Nonmetastatic HER2-positive BC	-	236 (BC recurrence=66)	Wine consumption (>2 drinks/week vs. 0-2 drinks/week)	Disease-free survival	0.42 (0.18 to 0.95)	age at diagnosis, BMI, stage, adjuvant endocrine therapy, and radiotherapy
								Beer consumption (>2 drinks/week vs. 0-2 drinks/week)		1.60 (0.47 to 5.47)	
2018 Madden JM	The National cancer registry Ireland (NCRI; mean 2.3 years)	Ireland	Postdiagnostic	-	I-III	Mean 67 years	5,417 (BC-specific death =806)	Vitamin D supplements (User vs. Non-user)	Breast cancer-specific mortality	0.80 (0.64 to 0.99)	age at diagnosis, smoking status, comorbidity score, tumour stage, tumour grade, ER, PR and HER 2-receptor status, bisphosphonate in year prior and post diagnosis, chemotherapy, anti-oestrogen therapy, NSAID, statins, anti-diabetic medication use
2019 Minami Y	Miyagi cancer center hospital (MCCH; median 8.6 years)	Japan	Prediagnostic	Premenopausal + Postmenopausal	I-IV	Mean 57.2 years	1,420 (Total death =261; BC-specific	Alcohol drinking (Current vs. Never)	All-cause mortality	0.76 (0.53 to 1.08)	age, year of diagnosis, stage, hormone receptor, menopausal status, referral
								Alcohol drinking	Breast cancer-specific mortality	0.70 (0.47 to 1.04)	

Study	Project (follow-up period)	Countries	Diagnostic	Menopausal status	BC stage	Age	Sample size (n)	Exposures	Outcomes	HR/RR (95% CIs)	Covariates
							death =193)	(Current vs. Never)			status, radiation therapy, chemotherapy, endocrine therapy, family history of breast cancer in patients or siblings, smoking, BMI, physical activity, comorbidities, occupation, age at menarche, parity history, menopausal status, and intake of folate and energy
2020 Anderson JLM	The Danish diet, cancer and health cohort (DCH; median 7 years)	Denmark	Prediagnostic	Postmenopausal	-	Mean 66 years	1,965 (Total death =460; BC-specific death =301; BC recurrence =309)	Total whole grain products (50 g/day)	All-cause mortality	1.02 (0.95 to 1.11)	age at diagnosis, year at diagnosis, time of follow-up since diagnosis, alcohol, smoking, physical activity, BMI, educational level, tumor size, nodal status, ER status
								Rye bread (50 g/day)		1.10 (0.98 to 1.25)	
								Whole grain bread (40 g/day)		1.02 (0.93 to 1.11)	
								Oatmeal/muesli (50 g/day)		0.80 (0.61 to 1.05)	
								Total dairy products (200 g/day)		1.02 (0.96 to 1.08)	
								Milk (200 g/day)		1.03 (0.96 to 1.10)	
								Yogurt		0.90	

Study	Project (follow-up period)	Countries	Diagnostic	Menopausal status	BC stage	Age	Sample size (n)	Exposures	Outcomes	HR/RR (95% CIs)	Covariates
								(200 g/day)		(0.75 to 1.07)	
								Cheese (50 g/day)		1.16 (0.97 to 1.39)	
								Total whole grain products (50 g/day)	Breast cancer-specific mortality	1.06 (0.97 to 1.17)	
								Rye bread (50 g/day)		1.11 (0.96 to 1.29)	
								Whole grain bread (40 g/day)		1.04 (0.94 to 1.15)	
								Oatmeal/muesli (50 g/day)		0.99 (0.73 to 1.35)	
								Total dairy products (200 g/day)		0.98 (0.91 to 1.06)	
								Milk (200 g/day)		0.99 (0.91 to 1.08)	
								Yogurt (200 g/day)		0.86 (0.69 to 1.08)	
								Cheese (50 g/day)		1.18 (0.94 to 1.47)	
								Total whole grain products (50 g/day)		1.03 (0.94 to 1.13)	
								Rye bread (50 g/day)		1.04 (0.89 to 1.20)	
								Whole grain bread (40 g/day)	Breast cancer recurrence	1.04 (0.94 to 1.15)	
								Oatmeal/muesli (50 g/day)		0.90 (0.66 to 1.22)	
								Total dairy products (200 g/day)		0.98 (0.91 to 1.06)	

Study	Project (follow-up period)	Countries	Diagnostic	Menopausal status	BC stage	Age	Sample size (n)	Exposures	Outcomes	HR/RR (95% CIs)	Covariates
								Milk (200 g/day)		0.96 (0.89 to 1.05)	
								Yogurt (200 g/day)		1.02 (0.83 to 1.27)	
								Cheese (50 g/day)		1.17 (0.94 to 1.45)	
			Postdiagnostic				977 (Total death - 175; BC-specific death =121; BC recurrence =152)	Total whole grain products (50 g/day)	All-cause mortality	0.99 (0.88 to 1.12)	
								Rye bread (50 g/day)		1.21 (0.98 to 1.47)	
								Whole grain bread (40 g/day)		0.95 (0.81 to 1.10)	
								Oatmeal/muesli (50 g/day)		0.75 (0.53 to 1.07)	
								Total dairy products (200 g/day)		0.99 (0.90 to 1.09)	
								Milk (200 g/day)		1.00 (0.90 to 1.12)	
								Yogurt (200 g/day)		0.93 (0.74 to 1.16)	
								Cheese (50 g/day)		1.09 (0.70 to 1.47)	
								Total whole grain products (50 g/day)	Breast cancer-specific mortality	1.05 (0.92 to 1.21)	
								Rye bread (50 g/day)		1.34 (1.06 to 1.70)	

								Whole grain bread (40 g/day)		0.97 (0.81 to 1.15)	
								Oatmeal/muesli (50 g/day)		0.82 (0.55 to 1.22)	
								Total dairy products (200 g/day)		0.99 (0.87 to 1.12)	
								Milk (200 g/day)		1.02 (0.89 to 1.17)	
								Yogurt (200 g/day)		0.84 (0.60 to 1.18)	
								Cheese (50 g/day)		0.95 (0.66 to 1.37)	
								Total whole grain products (50 g/day)	Breast cancer recurrence	0.98 (0.83 to 1.15)	
								Rye bread (50 g/day)		1.27 (0.97 to 1.66)	

Study	Project (follow-up period)	Countries	Diagnostic	Menopausal status	BC stage	Age	Sample size (n)	Exposures	Outcomes	HR/RR (95% CIs)	Covariates
								Whole grain bread (40 g/day)		0.83 (0.66 to 1.04)	
								Oatmeal/muesli (50 g/day)		0.93 (0.62 to 1.40)	
								Total dairy products (200 g/day)		0.93 (0.80 to 1.07)	
								Milk (200 g/day)		0.92 (0.78 to 1.08)	
								Yogurt (200 g/day)		0.92 (0.67 to 1.25)	
								Cheese (50 g/day)		1.23 (0.85 to 1.78)	

2020 Madden JM	National cancer registry Ireland (NCRI; median 4.2 years)	Ireland	Postdiagnostic	-	I-III	Mean 67 years	2,570 (Total death =386)	Vitamin D supplements (User vs. Non-user)	All-cause mortality	0.84 (0.73 to 0.99)	Age, smoking status, comorbidity score, tumor stage and grade, estrogen receptor, progesterone receptor, HER2 status, use of nonsteroidal antiinflammatory drugs, statins, antidiabetic medication during the year before

Study	Project (follow-up period)	Countries	Diagnostic	Menopausal status	BC stage	Age	Sample size (n)	Exposures	Outcomes	HR/RR (95% CIs)	Covariates
											diagnosis, receipt of chemotherapy, antiestrogen therapy during the year after diagnosis, bisphosphonate use during the year before diagnosis, bisphosphonate use after diagnosis
2020 Wang F	Shanghai breast cancer survival study (SBCSS; median 5.3 years)	China	Postdiagnostic	Premenopausal + Postmenopausal	I-IV	Mean 58.9 years	3,450 (Total death =374; BC-	CHFP-2007 (Q4 vs. Q1)	All-cause mortality	0.66 (0.48 to 0.89)	Age, total energy intake, income, education, marriage,
								CHFP-2016 (Q4 vs. Q1)		0.75 (0.55 to 1.01)	

							specific death =228)	DASH (Q4 vs. Q1)		0.66 (0.49 to 0.91)	menopausal status at diagnosis, BMI, physical activity, ER, PR, HER2, TNM stages, comorbidity,
								HEI-2015 (Q4 vs. Q1)		0.79 (0.57 to 1.10)	
								CHFP-2007 (Q4 vs. Q1)	Breast cancer-specific mortality	0.64 (0.44 to 0.93)	
								CHFP-2016 (Q4 vs. Q1)		0.67 (0.45 to 0.99)	
								DASH (Q4 vs. Q1)		0.60 (0.40 to 0.90)	
								HEI-2015 (Q4 vs. Q1)		0.89 (0.59 to 1.33)	

Study	Project (follow-up period)	Countries	Diagnostic	Menopausal status	BC stage	Age	Sample size (n)	Exposures	Outcomes	HR/RR (95% CIs)	Covariates
											chemotherapy, radiotherapy, immunotherapy
2020 Wang K	The prostate, lung, colorectal, and ovarian cancer screening (PLCO; median 14.6 years)	United States	Postdiagnostic	Postmenopausal	I-IIIA	Mean 65 years	1,064 (Total death =296; BC-specific death =100)	E-DII (T3 vs. T1)	All-cause mortality	1.34 (1.01 to 1.81)	Age at diagnosis, years from breast cancer diagnosis to DHQ completion, stage, adjusted for total energy intake, BMI, trial arm, race, marital status, income, educational level, smoking status, HRT, history of diabetes, physical activity, estrogen receptor status, progesterone receptor status
								E-DII (T3 vs. T1)	Breast cancer-specific moratlity	1.47 (0.89 to 2.43)	

Study	Project (follow-up period)	Countries	Diagnostic	Menopausal status	BC stage	Age	Sample size (n)	Exposures	Outcomes	HR/RR (95% CIs)	Covariates
2020 Wu T	Women's healthy eating and living (WHEL; median 7.3 years)	United States	Prediagnostic + Postdiatnostic	Premenopausal + Postmenopausal	I-IIIA	Mean 50 years	2,950 (Total death =295; BC-specific death =249; BC recurrence=490)	PRAL (Q4 vs. Q1)	All-cause mortality	1.30 (0.87 to 1.94)	age at dianosis, race/ethnicity, education level, intervention group, menopausal status at baseline, total calorie intake, alcohol intake, physical activity, BMI, number of comorbidities, tumor stage, tumor size, estrogen and progesterone receptor status, tamoxifen use, radiotherapy, and chemotherapy
								NEAP (Q4 vs. Q1)		1.54 (1.04 to 2.29)	
								PRAL (Q4 vs. Q1)	Breast cancer-specific mortality	1.27 (0.83 to 1.94)	
								NEAP (Q4 vs. Q1)		1.52 (1.01 to 2.32)	
								PRAL (Q4 vs. Q1)	Breast cancer recurrence	1.09 (0.83 to 1.43)	
								NEAP (Q4 vs. Q1)		1.15 (0.88 to 1.50)	

Abbreviations: BC, breast cancer; BMI, body mass index; HRT, hormone replacement therapy; ER, estrogen receptor; PR, progesterone receptor; TNM, tumor-nodes-metastases; HEI, health eating index; AHEI, alternate health eating index; DQIR, diet quality index-revised; RFS, recommended food score; aMED, alternate mediterranean diet score; WCRF/AICR, world cancer research fund and the American institute for cancer research; DASH, dietary approaches to stop hypertension; AJCC, the American joint committee on cancer; ACS, American cancer society; MET, metabolic equivalent of task; DII, dietary inflammatory index; CHFP, Chinese food pagoda; E-DII, energy-adjusted dietary inflammatory index; PRAL, the potential renal acid load; NEAP, the net endogenous acid production.

Table S2. NewCastle-Ottawa quality assessment scale for cohort studies in the systematic review and meta-analysis.

	Selection				Comparability		Outcomes			Total
Study	Representativeness of the exposed cohort	Selection of the nonexposed cohort	Ascertainment of exposure	Outcome of interest was	Age or gender	Additional factors	Assessment of outcome	Follow-up length	Adequacy of follow-up of cohorts	

				not present at start						
1991 Ewertz M	1	1	0	1	1	1	1	1	1	8
1993 Rohan TE	1	1	1	1	1	1	1	1	0	8
1994 Jain M	1	1	1	1	1	1	1	1	1	9
1998 Hebert JR	1	1	0	1	1	1	1	1	1	8
1999 Holmes MD	0	1	0	1	1	1	1	1	1	7
2003 Fleischauer AT	1	1	0	1	1	1	1	1	1	8
2004 Borugain MJ	1	1	0	1	1	1	1	1	1	8
2005 Boyapati SM	1	1	1	1	1	1	1	1	0	8
2005 Kroenke CH	0	1	0	1	1	1	1	1	0	6
2006 Cui Y	1	1	1	1	1	1	1	0	0	7
2006 McEligot AJ	1	1	0	1	1	1	1	1	1	8
2006 Fink BN	1	1	1	1	1	1	1	1	1	9
2007 Fink BN	1	1	1	1	1	1	1	1	1	9
2008 Xu X	1	1	1	1	1	1	1	1	0	8
2009 Guha N	1	1	0	1	1	1	1	1	1	8
2009 Kwan ML	1	1	0	1	1	1	1	1	0	7
2009 Shu XO	1	1	1	1	1	1	1	0	1	8
2010 Hellmann SS	1	1	1	1	1	1	1	1	1	9
2010 Kang X	1	1	1	1	1	1	1	1	0	8
2010 Kwan ML	1	1	0	1	1	1	1	1	0	7
2011 Beasley JM	1	1	0	1	1	1	1	1	0	7
2011 Belle FN	1	1	1	1	1	1	1	1	1	9
2011 Caan BJ	1	1	0	1	1	1	1	1	0	7
2011 George SM	1	1	0	1	0	1	1	1	1	7
2011 Kim EHJ	0	1	0	1	1	1	1	1	1	7
2011 Kwan ML	1	1	0	1	1	1	1	1	1	8
2011 Nechuta S	1	1	1	1	1	1	1	0	0	7
2011 Saquib J	1	1	0	1	1	1	1	1	0	7
2012 Greenlee H	1	1	0	1	1	1	1	1	0	7
2012 Harris HR(a)	1	1	0	1	1	1	1	1	0	7
2012 Harris HR(b)	1	1	0	1	1	1	1	1	0	7
2012 Harris HR(c)	1	1	0	1	1	1	1	1	0	7

2012 Nechuta SJ	1	1	0	1	1	1	1	1	1	8
2012 Vrieling A	1	1	0	1	1	1	1	1	1	8
2013 Conroy SM	1	1	0	1	1	1	1	1	1	8
2013 Holm M	1	1	0	1	0	1	1	1	1	7
	Selection				Comparability		Outcomes			Total
Study	Representativeness of the exposed cohort	Selection of the nonexposed cohort	Ascertainment of exposure	Outcome of interest was not present at start	Age or gender	Additional factors	Assessment of outcome	Follow-up length	Adequacy of follow-up of cohorts	
2013 Inoue-Choi M	1	1	0	1	1	1	1	1	0	7
2013 Izano MA	0	1	0	1	1	1	1	1	1	7
2013 Kroenke CH	1	1	0	1	1	1	1	1	0	7
2013 Kwan ML	1	1	0	1	1	1	1	1	1	8
2013 Newcomb PA	1	1	1	1	1	1	1	1	1	9
2013 Poole EM	1	1	0	1	1	1	1	0	1	7
2013 Vrieling A	1	1	1	1	1	1	1	1	1	9
2014 George SM	1	1	1	1	1	1	1	1	1	9
2015 Bao PP	1	1	1	1	1	1	1	1	0	8
2015 Jeffreys M	0	1	1	1	1	1	1	0	0	6
2015 Kyro C	1	1	0	1	1	1	1	1	0	7
2016 McCullough ML	1	1	0	1	1	1	1	1	0	7
2016 Neuhouser ML	1	1	1	1	0	1	1	0	1	7
2016 Tao MH	1	1	1	1	1	1	1	1	0	8
2017 Holmes MD	0	1	0	1	1	1	1	1	1	7
2017 Zeinomar N	0	1	1	1	1	1	1	1	1	8
2017 Zhang FF	0	1	0	1	1	1	1	1	0	6
2017 Zucchetto A	0	1	1	1	1	1	0	1	0	6
2018 Furrer D	1	1	0	1	1	1	1	1	1	8
2018 Madden JM	1	1	1	1	1	1	1	0	0	7
2019 Minami Y	1	1	0	1	1	1	1	1	1	8
2020 Anderson JLM	1	1	0	1	1	1	1	1	1	8
2020 Madden JM	1	1	1	1	1	1	1	0	0	7
2020 Wang F	1	1	1	1	1	1	1	1	1	9
2020 Wang K	1	1	0	1	1	1	1	1	1	8

2020 Wu T	1	1	1	1	1	1	1	1	1	9
